# Genome-Wide Identification and Expression Analysis of RLCK-VII Subfamily Genes Reveal Their Roles in Stress Responses of Upland Cotton

**DOI:** 10.3390/plants12173170

**Published:** 2023-09-04

**Authors:** Yuhan Cen, Shiyi Geng, Linying Gao, Xinyue Wang, Xin Yan, Yuxia Hou, Ping Wang

**Affiliations:** 1 Innovation Center of Pesticide Research, Department of Applied Chemistry, College of Science, China Agricultural University, Beijing 100193, Chinagengsy202309@163.com (S.G.);; 2 Key Laboratory of National Forestry and Grassland Administration on Pest Chemical Control, China Agricultural University, Beijing 100193, China

**Keywords:** cotton, RLCK-VII, phylogeny, expression, *Verticillium dahliae*

## Abstract

Receptor-like cytoplasmic kinase VII (RLCK-VII) subfamily members are vital players in plant innate immunity and are also involved in plant development and abiotic stress tolerance. As a widely cultivated textile crop, upland cotton (*Gossypium hirsutum*) attaches great importance to the cotton industry worldwide. To obtain details of the composition, phylogeny, and putative function of RLCK-VII genes in upland cotton, genome-wide identification, evolutionary event analysis, and expression pattern examination of RLCK-VII subfamily genes in *G. hirsutum* were performed. There are 129 RLCK-VII members in upland cotton (*GhRLCKs*) and they were divided into nine groups based on their phylogenetic relationships. The gene structure and sequence features are relatively conserved within each group, which were divided based on their phylogenetic relationships, and consistent with those in *Arabidopsis*. The phylogenetic analysis results showed that RLCK-VII subfamily genes evolved in plants before the speciation of *Arabidopsis* and cotton, and segmental duplication was the major factor that caused the expansion of *GhRLCKs.* The diverse expression patterns of *GhRLCKs* in response to abiotic stresses (temperature, salt, and drought) and *V. dahliae* infection were observed. The candidates that may be involved in cotton’s response to these stresses are highlighted. *GhRLCK7* (*GhRLCK7A* and *D*), which is notably induced by *V. dahliae* infection, was demonstrated to positively regulate cotton defense against *V. dahliae* by the loss-of-function assay in cotton. These findings shed light on the details of the RLCK-VII subfamily in cotton and provide a scaffold for the further function elucidation and application of *GhRLCKs* for the germplasm innovation of cotton.

## 1. Introduction

Cotton is a crucial economic crop and plays a significant role in the global textile industry. The cotton genus (*Gossypium* spp.) contains 41–47 diploid species and seven allotetraploid species [1,2]. *G. hirsutum* (AD_1_) is among the allotetraploid species and accounts for more than 95% of commercial cotton production worldwide [3]. Ancestral hybridization between A-genome progenitors from the Old World and D-genome ancestors from the New World, and subsequent chromosome doubling, resulted in the formation of allopolyploid cotton approximately 1–2 million years ago [4,5]. The completion of sequencing work on diploid cotton *G. raimondii* (D_5_) [6] and *G. arboreum* (A_2_) [7], as well as allotetraploid cotton *G. hirsutumtm* (AD_1_) [8,9] and *G. barbadense* (AD_2_) [10], has provided crucial references for exploring gene function and evolution at the whole-genome level.

Cotton plants are subjected to various biotic and abiotic stresses during their growth and development, especially drought, salinity, and wilt diseases, posing significant threats to the sustainable development of the cotton industry worldwide [11,12]. Among the different stresses, the occurrence of Verticillium wilt has become increasingly severe due to long-term continuous cropping, returning cotton straw to the field, and relative lag in updates in resistant germplasm resources [13,14]. Therefore, there is an urgent need to generate sustainable and stress-tolerant cotton varieties. In the last decade, with the release of genomic data from different cotton species, functional genomics studies of cotton have become increasingly in-depth and have greatly boosted the identification of stress-related genes in cotton germplasms, laying the foundation for the innovation of elite cotton varieties via modern genetic engineering approaches [15].

Receptor-like cytoplasmic kinases (RLCKs) are the central players in plant signaling pathways and regulate various cellular events during defense against the biotic and abiotic stresses, growth, development, and reproduction of plants through associations with corresponding receptor-like kinases (RLKs) and substrates [16,17]. There is a significant abundance of RLCKs in plants, and 149 and 379 RLCKs have been identified in the *Arabidopsis* and rice genomes, respectively [18,19]. The RLCKs in plants were divided into 17 subgroups and named RLCK-II and RLCK-IV~RLCK-XIX [18]. Among them, RLCK-VII subfamily members have a conserved role as convergent substrates of cell membrane-localized receptor-like kinases (RLKs) and regulate multiple signaling nodes, orchestrating a complex array of defense responses against phytopathogens [20]. The most representative one is BOTRYTIS-INDUCED KINASE1 (BIK1). Along with its close homolog PBS1-Like1 (PBL1), BIK1 mediates several pathogen-associated molecular patterns (PAMPs), eliciting defense responses including calcium influx, ROS burst, actin filament bundling, callose deposition, stomatal closure, and seedling growth inhibition by associating directly with a few RLKs, such as FLS2, EFR, CERK1, and PEPR1 in *Arabidopsis* [17]. Various members of the RLCK-VII subgroups have been employed by the same or different RLKs to regulate downstream immune responses, like PCRK1 [21] and PCRK2 [22], PBL27 [23], PBL19 [24], and RIPK in *Arabidopsis* [25]; CaPIK1 in pepper [26]; and OsRLCK176 [27], OsRLCK118 [28], and OsRLCK185 [29] in rice, which mediates one or multiple defense responses in response to pathogen invasion. Meanwhile, there are also several RLCK-VII members involved in plant abiotic stress tolerance, for example, *OsGUDK* from rice [30,31] and Esi47 from barley [32]. However, none of RLCK-VII members have been characterized in cotton, and the family composition, molecular evolution, and resistance function of RLCK-VII subfamily genes in cotton are still elusive. Thus, the disclosure of the RLCK-VII members in cotton will be critical for the mining of valuable gene resources for the innovation of elite cotton varieties in the face of ever-changing environments and pathogens.

In this study, we conducted a genome-wide screen of the RLCK-VII subfamily genes in *G. hirsutum* (*GhRLCK*). The characterization and evolution analysis of 139 *GhRLCKs* were performed based on their gene structures, chromosomal localizations, cladograms, and gene syntenies. The involvement of *GhRLCKs* in response to abiotic stresses (drought, cold, and heat) and biotic stress (*V. dahliae* infection) was investigated. Furthermore, the VIGS approach was employed to verify the role of the representative gene *GhRLCK7* in upland cotton’s defense against *V. dahliae*. This study sheds light on the mystery of RLCK VII subfamily genes in cotton and lays the foundation for the further function elucidation of these genes in the broad tolerance of cotton to stresses.

## 2. Result

### 2.1. Identification of RLCK-VII Subfamily Genes in Gossypium hirsutum

**Figure 1:**
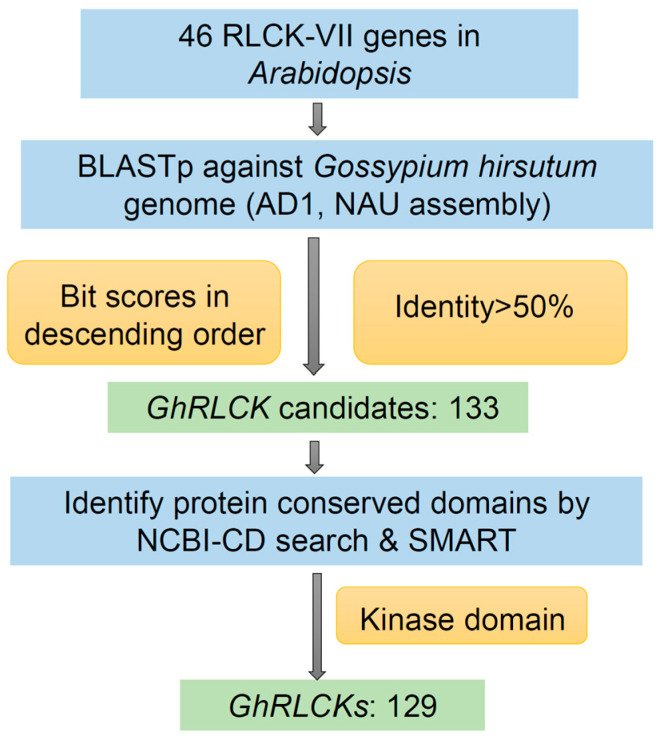
Genome-wide identification pipeline of *GhRLCKs* in *Gossypium hirsutum*. Forty-six *Arabidopsis* RLCK-VII protein sequences were used as templates for BLASTp against *G. hirsutum* genome (AD1, NAU assembly) in CottonFGD. *GhRLCK* candidates were further refined using protein domain prediction tools (NCBI-CD search and SMART) to eliminate non-RLCKs by manually checking the protein motifs.

In order to unravel the RLCK-VII subfamily of upland cotton (*G. hirsutum*), we performed a systematic identification of cotton RLCK -VII genes (*GhRLCKs*) from the genome of *G. hirsutum* (Figure 1). Through BLASTp searches using 46 AtRLCKs as queries (Table S1) and the removal of redundant genes, 133 *GhRLCKs* were identified from the whole genome of upland cotton. Four genes were eliminated from the list due to the presence of extra protein motifs relative to RLCKs in *Arabidopsis*, which were double-checked using NCBI-CDD and SMART. A total of 129 *GhRLCKs* were identified from *G. hirsutum*; in other words, there are 129 genes in the RLCK VII subfamily in upland cotton (Table S2). We named the individual *GhRLCKs* following the previous naming scheme [33] (Table 1). The coding sequence lengths of the 129 *GhRLCK* genes range from 1221 bp (*GhRLCK19D*) to 7322 bp (*GhRLCK58A*). The exon number of most *GhRLCKs* ranges from four to seven, but *GhRLCK19D* has two exons. The *GhRLCK* proteins contain 323–569 aa with molecular weights ranging from 35.91 kDa (*GhRLCK9A*) to 61.69 KDa (*GhRLCK17A*). The isoelectric points of these proteins range from 3.39 (*GhRLCK48D*) to 9.789 (*GhRLCK62A*), the instability indexes range from 23.98 (*GhRLCK15D*) to 63.01 (*GhRLCK19D*), and the aliphatic indexes are in the range of 60.08 (*GhRLCK19D*) to 93.62 (*GhRLCK28D*) (Table 1).

### 2.2. Phylogenetic Analysis of RLCK VII Subfamily Genes in Cotton and Arabidopsis

**Figure 2:**
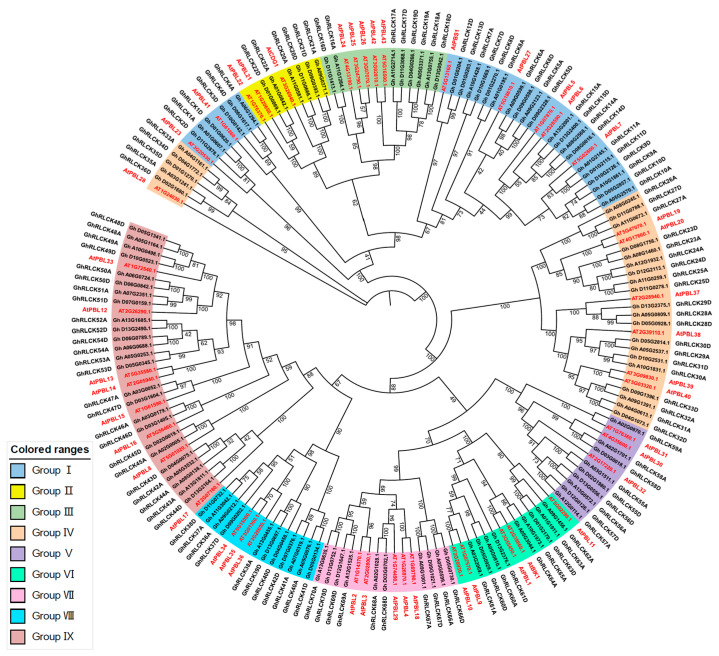
Phylogenetic analysis of RLCK-VII family members from *G. hirsutum* and *A. thaliana*. The phylogenetic tree was constructed using the protein sequences of 46 genes from *A. thaliana* and 129 genes from *G. hirsutum*. These genes were divided into 9 groups (Group I-IX) and are indicated by different colors. The genes from *A. thaliana* are labeled in red.

To elucidate the evolutionary relationship between RLCK-VII subfamily genes in *A. thaliana* and *G. hirsutum,* A rootless phylogenetic tree was constructed with 129 *GhRLCKs*, and 46 homologs in *Arabidopsis* (Figure 2). Based on the 46 RLCK-VII subfamily categories from *Arabidopsis* [20], the GhRLCKs were divided into nine groups (Group I to Group IX), in which Group I and Group II are the largest and smallest groups with 26 and 6 members, respectively. Group III, Group IV, Group V, Group VI, Group VII, Group VII, and Group IX contain 8, 25, 9, 9, 10, 11, and 25 genes, respectively (Figure 2). The above results suggest that RLCK-VII subfamily genes evolved before cotton and *Arabidopsis* speciation and were unevenly distributed in all groups of cotton.

### 2.3. Conserved Domains and Structure Analysis of RLCK-VII Genes in G. hirsutum and A. thaliana

**Figure 3:**
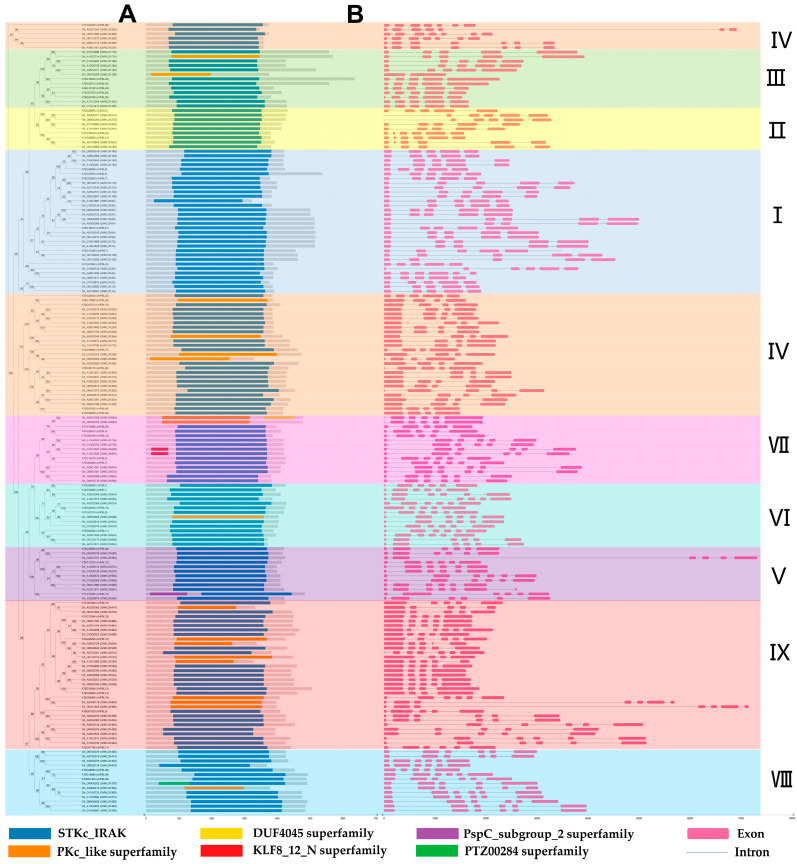
Dendrogram, gene structure, and conserved domain of RLCK- VII subfamily genes. (**A**) The conserved domains of these genes were predicted using NCBI-CDD. The protein domain schematics are included at the bottom. (**B**) Exon–intron structure. Pink boxes and grey horizontal lines represent exons and introns, respectively. Different groups are indicated by different colors and the group numbers are shown on the right.

To further understand the conservation and divergence of the structures and domains of RLCK-VII subfamily members from *G. hirsutum* and *A. thaliana*, the conserved domains and gene structures of the RLCK-VII genes were analyzed. The prediction of conserved domains showed that all the proteins contain either the STKc-IRAK- or PKc-like superfamily domain (Figure 3A). The analysis of gene structure revealed that the amounts of exons vary between four and seven, with four exons in *GhRLCK19D*, and seven exons in *GhRLCK46A*, *GhRLCK55A*, *GhRLCK33A*, *GhRLCK35A*, *GhRLCK51A*, *GhRLCK35D*, *GhRLCK36D*, *GhRLCK46D*, and *GhRLCK34D* (Figure 3B). Overall, most genes within the same group exhibit similar gene sizes and structures, implying that the same group members may share similar functions.

### 2.4. Chromosomal Location, Gene Duplication, and Synteny Analysis of GhRLCKs

The physical locations of *GhRLCKs* on chromosomes were mapped to investigate their distributions in the *G. hirsutum* genome. Among these genes, eight genes Gh_A01G2145 (*GhRLCK11A*), Gh_A07G2351 (*GhRLCK51A*), Gh_A08G2568 (*GhRLCK14A*), Gh_A11G3042 (*GhRLCK37A*), Gh_A03G2069 (*GhRLCK61A*), Gh_D01G2270 (*GhRLCK8D*), Gh_D10G2531 (*GhRLCK31D*) and Gh_D13G2490 (*GhRLCK52D*) are localized on scaffolds and 121 genes are randomly distributed on 26 chromosomes with 59 members in the At sub-genome and 62 members in Dt sub-genome (Figure S1). The numbers of genes on the A01 to A13 chromosomes are 4, 4, 4, 2, 11, 3, 1, 3, 6, 5, 5, 3, and 7, respectively. The gene numbers on D01 to D13 chromosomes is 7, 3, 4, 5, 8, 3, 2, 2, 6, 4, 7, 3, and 8. The A07 chromosome contains the least genes and the A05 chromosome contains the most genes. The duplication of genes through events such as whole-genome, segmental, dispersed, or tandem duplication is widely recognized as a key driving force in the process of evolution [34]. Based on the evolution event analyses of all these genes, the tandem arranged genes Gh-D01G0203 and Gh-D01G0204 (*GhRLCK13D* and *GhRLCK12D*) on chromosome D01 represent the only tandem duplication event in *GhRLCK* expansion during the speciation of allotetraploid upland cotton.

**Figure 4:**
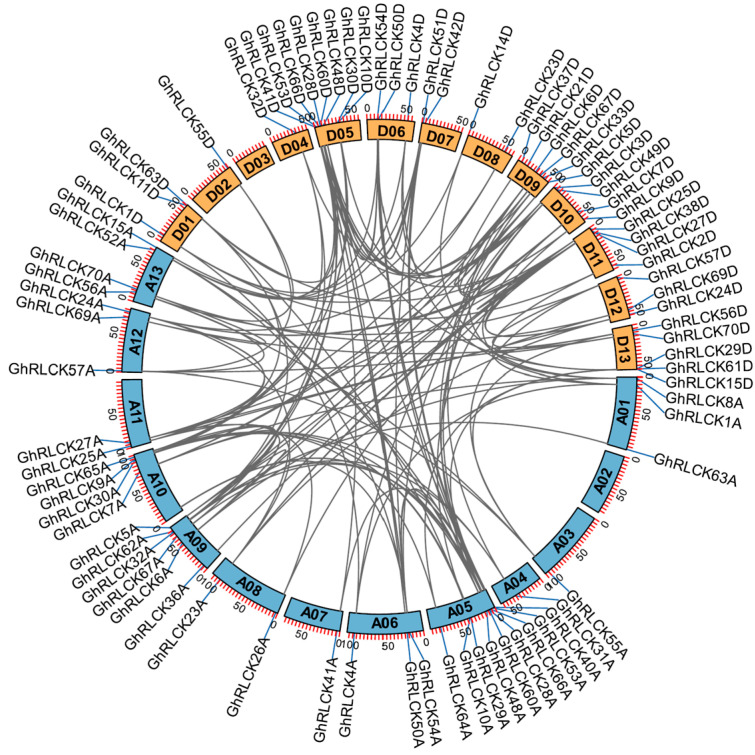
The syntenic analysis of *GhRLCK* members. The relationship is presented using Circos software. The paralogous gene pairs are linked with gray lines. Chromosomes from the At and Dt sub-genomes are indicated in blue and orange.

To further explore the locus relationship between paralogous gene pairs of the At and Dt sub-genomes, synteny analysis was performed and 135 paralogous gene pairs were identified in *G. hirsutum* (Table S3), which excluded the homoeologous gene pairs in allotetraploid cotton. Among these, 35 paralogous gene pairs were found in the At sub-genome; 32 paralogous gene pairs were present in Dt sub-genome; 68 paralogous gene pairs were found between At and Dt sub-genome. Most genes undergo multiple segmental duplications (Figure 4). The mechanisms driving gene replication in *G. hirsutum* indicated that most *GhRLCKs* evolved through segmental duplications of other members, whereas only *GhRLCK13D* and *GhRLCK12D* were derived from tandem chromosomal distribution.

The selective pressures were further investigated to evaluate the interrelationships and evolutionary dynamics among paralogous genes. The *Ka*/*Ks* ratios of two protein-coding genes were employed to evaluate the presence of selective pressure [35]. *Ka*/*Ks* ratios of 1.0 specify pseudogenes produced after neutral selection, while *Ka*/*Ks* ratios < 1.0 demonstrate the capacity of duplicated genes for purifying selection; however, *Ka*/*Ks* ratios > 1.0 indicate positive selection [36]. Herein, the *Ka*, *Ks*, and *Ka*/*Ks* values among *GhRLCKs* were calculated and the results showed that all of the duplicated gene pairs had a *Ka*/*Ks* ratio of less than 1.0 (around 0.3) (Table S4), suggesting that these genes experienced strong purifying selective pressures. These observations indicated that the upland cotton *GhRLCKs* were prone to synonymous mutations during speciation, leading to function conservation of the subfamily.

### 2.5. Tissue- and Organ-Specific Expression Profiling of GhRLCKs in Upland Cotton

In order to provide hints about *GhRLCKs*’ potential roles in upland cotton, the tissue- and organ-specific expression levels of *GhRLCKs* were analyzed based on the published RNA-seq dataset of *G. hirsutum* [37]. The results showed that *GhRLCKs* exhibited different expression patterns in leaf, stem, root, petal, anther, bract, filament, pistil, sepal, torus, ovule, and fiber tissues at 10 days post-anthesis (DPA) (Figure S2). Several genes, such as *GhRLCK60D*, *GhRLCK23D*, and *GhRLCK23A*, exhibited abundant expression in the petal, anther, sepal, bract, filament, pistil, and torus, suggesting that they may participate in cotton reproduction, while 23 *GhRLCK* genes (*GhRLCK18A*-*GhRLCK2D*) exhibited extremely low or no expression in the 12 tested tissues and organs (Figure S2). *GhRLCK66A* was extremely highly expressed in all tissues, implying its essential role during the whole growth period of cotton. It should be noted that *GhRLCK51A* and *D* exhibited a dominant expression pattern in the petal, anther, and filament. In addition, several genes, *GhRLCK50A*, *GhRLCK52A/D*, *GhRLCK70D*, and *GhRLCK46A*, were highly and specifically expressed in the root.

### 2.6. Expression Patterns of GhRLCK Genes in Upland Cotton under Abiotic Stresses

To further explore the roles of *GhRLCK* genes in the responses to abiotic stresses, the expression patterns of *GhRLCK* genes under cold (4 °C), heat (37 °C), salt (NaCl), and polyethylene glycol (PEG)-mimic drought treatments were examined.

**Figure 5:**
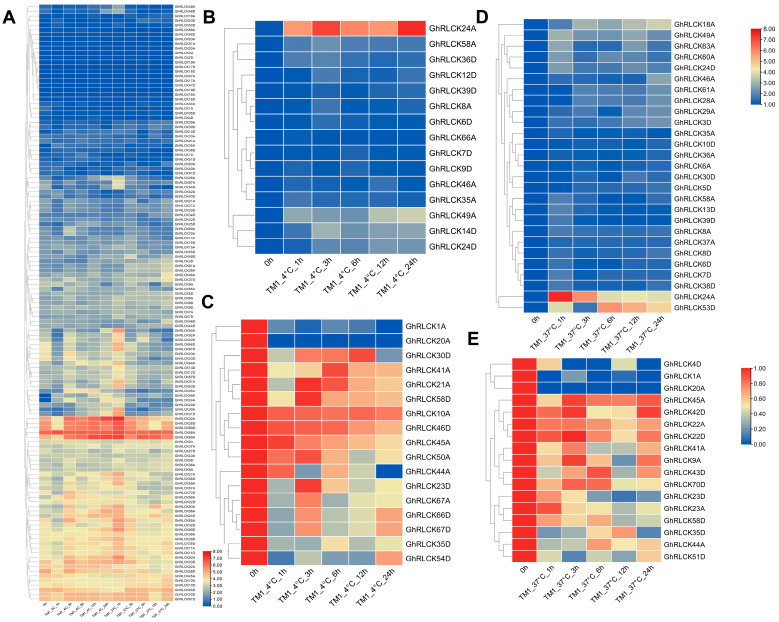
Expression profiles of *GhRLCK* genes in response to low (4 °C) and high (37 °C) temperatures. (**A**) Overview of expression abundances of *GhRLCKs* in response to low (4 °C) and high (37 °C) temperatures. Heatmap was generated based on FPKM values. (**B**) Upregulated genes upon 4 °C treatment. (**C**) Downregulated genes upon 4 °C treatment. (**D**) TE upregulated genes under 37 °C treatment. (**E**) Downregulated genes under 37 °C treatment. Heatmaps were generated based on relative expression levels (**B**–**E**). Scale bars are indicated on the left.

**Figure 6:**
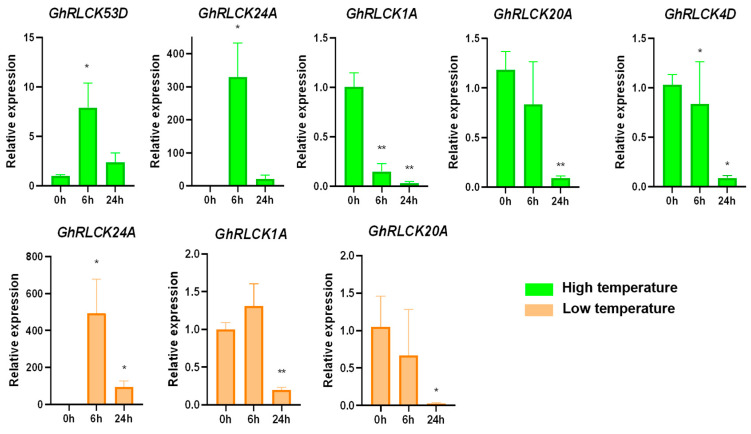
Expression levels of *GhRLCK* genes in response to low (4 °C) and high (37 °C) temperatures according to RT-qPCR analysis. Data are presented as mean ± SE from three independent repeats. Asterisks represent significant differences compared with results at 0 h according to two-tailed Student’s t-tests (* *p* < 0.05, ** *p* < 0.01).

Under hot and cold conditions, 129 *GhRLCKs* showed different expression levels (Figure 5). A portion of the *GhRLCKs* (*GhRLCK48A–GhRLCK41D*) exhibited low expression abundances under both treatments. Five genes (*GhRLCK32A*, *GhRLCK28D*, *GhRLCK60A* and *D*, and *GhRLCK66A*) exhibited high expression levels in all the samples (Figure 5A). Thirty-one of the *GhRLCKs*, including *GhRLCK26A*, *GhRLCK67A*, *GhRLCK54D*, etc., exhibited impaired expression levels 1 h after cold stress, whereas they showed upregulated expression levels 1 h after heat stress. Upon cold treatment, 32 *GhRLCKs* showed altered expression compared to the results at 0 h. Fifteen *GhRLCKs* (*GhRLCK24A*, *GhRLCK58A*, etc.) were persistently upregulated at 1, 3, 6, 12, and 24 h of cold stress (Figure 5B) and 17 genes showed decreased expression consistently at all five time points after cold treatment (Figure 5C). Remarkably, *GhRLCK24A* was notably upregulated at all time points after treatment compared with the results at 0 h (Figure 5B), indicating the pivotal role of *GhRLCK24A* in the cold response of cotton. Meanwhile, *GhRLCK1A* and *GhRLCK20A* were significantly suppressed at 1–24 h of cold treatment (Figure 5C). Therefore, the expression levels of *GhRLCK24A*, *GhRLCK1A*, and *GhRLCK20A* were further investigated via RT-qPCR analyses (Figure 6). The results showed that the expression of *GhRLCK24A* was significantly induced at 6 h and 24 h after cold treatment and the expression levels of *GhRLCK1A* and *GhRLCK24A* were remarkably downregulated at 24 h after cold treatment. These are largely consistent with the results from RNA-seq and indicate that their underlying roles deserve to be explored. Twenty-seven of the *GhRLCKs* (*GhRLCK18A–GhRLCK53D* exhibited continuously elevated expression levels (Figure 5D), and 17 of the *GhRLCKs* (*GhRLCK4D–GhRLCK51D*) were continuously downregulated in response to heat stress (Figure 5E). Among these genes, the expression levels of two genes (*GhRLCK24A and GhRLCK53D*) were remarkably elevated and those of three genes (*GhRLCK1A*, *GhRLCK20A*, *and GhRLCK4D*) were significantly reduced after 24 h of heat treatment. The expression levels of these five genes were further examined via RT-qPCR analyses and they were consistent with the results of RNA-seq (Figure 6), suggesting that they may be involved in cotton’s response to heat stress.

**Figure 7:**
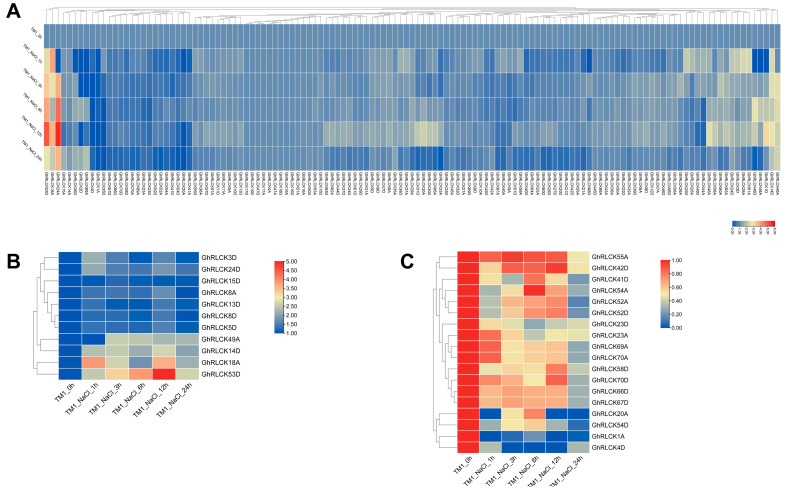
The expression patterns of *GhRLCKs* in response to salt treatment. (**A**) The overview of *GhRLCK* expression in response to NaCl treatment. The heatmap was generated based on the FPKM values. (**B**) The upregulated genes under NaCl treatment. (**C**) The downregulated genes under NaCl treatment. The heatmaps were generated based on the relative expression levels (**B**,**C**). The scale bars are presented adjacent to the charts.

**Figure 8:**
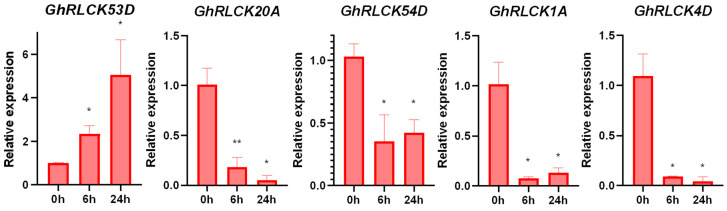
Expression levels of *GhRLCK* genes in response to salt treatment according to RT-qPCR analysis. Data are presented as mean ± SE from three independent repeats. Asterisks represent significant differences compared with results at 0 h according to two-tailed Student’s t-tests (* *p* < 0.05, ** *p* < 0.01).

*GhRLCKs* exhibited different expression patterns in response to NaCl treatment and the expression of the majority of the genes was low, except *GhRLCK24A*, *GhRLCK18A*, and *GhRLCK53D*, whose expression abundances were highly increased at one or more time points during NaCl treatment (Figure 7A). The expression levels of *GhRLCK1D* and *GhRLCK48A* and *D* were impaired at 1 h, and then, increased at 3, 6, and 12 h during NaCl treatment. Among the genes whose expression levels were sustainedly changed, 11 of the *GhRLCKs* (*GhRLCK3D–GhRLCK55D*) were upregulated (Figure 7B), and 18 of the *GhRLCKs* (*GhRLCK55A–GhRLCK4D*) were continuously downregulated (Figure 7C). Intriguingly, the expression levels of *GhRLCK53D*, *GhRLCK20A*, *GhRLCK54D*, *GhRLCK1A*, and *GhRLCK4D* were remarkably altered after NaCl treatment. Thus, their expressions were confirmed via RT-qPCR analysis and all five genes exhibited the same expression trends as those in RNAseq, namely, *GhRLCK53D* expression was notably induced whereas the expressions of others (*GhRLCK20A*, *GhRLCK54D*, *GhRLCK1A*, and *GhRLCK4D*) were significantly inhibited after NaCl treatment (Figure 8). Based on the data, more attention should be paid to the functional analysis of these genes in the salt response of cotton.

**Figure 9:**
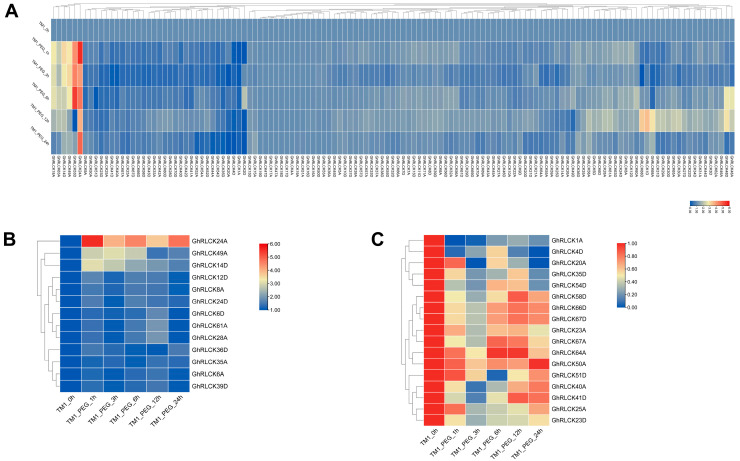
The expression patterns of *GhRLCKs* in response to PEG-mimic drought treatment. (**A**) An overview of *GhRLCK* expression in response to PEG. The heatmap was generated based on the FPKM values. (**B**) The upregulated genes under PEG treatment. (**C**) The downregulated genes under PEG treatment. The heatmaps were generated based on the relative expression levels (**B**,**C**). The scale bars are presented adjacent to the charts.

**Figure 10:**
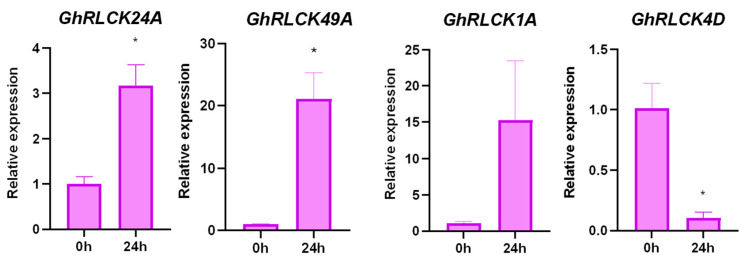
Expression levels of *GhRLCK* genes in response to PEG-mimic drought according to RT-qPCR analysis. Data are presented as mean ± SE from three independent repeats. Asterisks represent significant differences compared with results at 0 h according to two-tailed Student’s *t*-tests (* *p* < 0.05).

During PEG-simulated drought treatment, the expression abundances of *GhRLCKs* could be roughly divided into three categories: 41 of the *GhRLCK* genes (*GhRLCK35A–GhRLCK41A*) exhibited low expression abundances and showed almost no variations in expression level during PEG treatment (Figure 9A, right portion); 38 of the *GhRLCK* genes (*GhRLCK49A–GhRLCK15D*) had medium expression abundances (Figure 9A, middle part); and around 50 of the *GhRLCK* genes (*GhRLCK66A-GhRLCK58D*) showed high expression abundances, especially *GhRLCK66A* (Figure 9A, left part). There were 13 *GhRLCKs* whose expression levels were upregulated, particularly *GhRLCK24A* and *GhRLCK49A* (Figure 9B), and 17 of the *GhRLCKs* exhibited downregulated expression levels during PEG treatment, for example, *GhRLCK1A* and *GhRLCK4D* (Figure 9C). The expression levels of these four genes were next validated via RT-qPCR analysis (Figure 10). Similar to those in RNA-seq, the expression levels of *GhRLCK24A* and *GhRLCK49A* were notably unregulated and the expression of *GhRLCK4D* was downregulated at 24 h post-cold treatment (Figure 10). However, the expression level of *GhRLCK4D* did not exhibit a significant change (Figure 10) and this is not in line with the results of RNA-seq.

### 2.7. Expression Patterns of GhRLCKs under V. dahliae Infection

**Figure 11:**
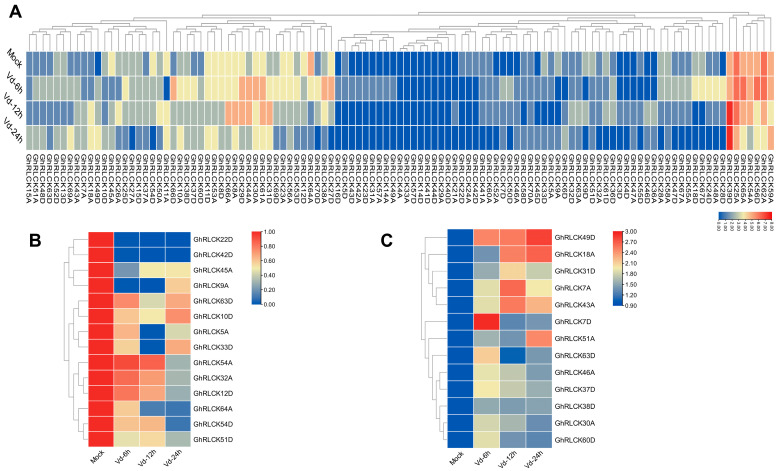
The expression levels of *GhRLCKs* in response to *V. dahliae* infection. (**A**) An overview of *GhRLCK* expression upon *V. dahliae* infection. The heatmap was generated based on the FPKM values. (**B**) The downregulated genes induced through *V. dahliae* inoculation. (**C**) The upregulated genes in response to *V. dahliae* infection. The heatmaps were generated based on the relative expression levels (**B**,**C**). The scale bars are presented adjacent to the charts.

**Figure 12:**
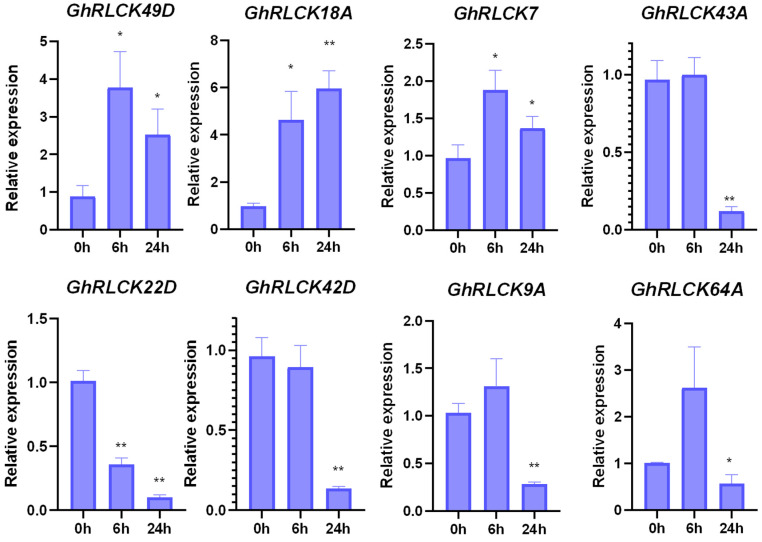
Expression levels of *GhRLCK* genes in response to *V.dahliae* infection according to RT-qPCR analysis. Data are presented as mean ± SE from three independent repeats. Asterisks represent significant differences compared with results at 0 h according to two-tailed Student’s *t*-tests (* *p* < 0.05, ** *p* < 0.01).

To uncover the disease-resistance function of *GhRLCKs* in upland cotton, we analyzed the expression patterns of *GhRLCKs* in response to the invasion of *V. dahliae*. Except for 20 *GhRLCKs* whose expression could not be detected, we obtained the expression patterns of 109 *GhRLCK* genes at 0 h, 6 h, 12 h, and 24 h post-inoculation (hpi) from the published transcriptome dataset [38]. The expression abundances of these genes were diverse. Among these genes, eight *GhRLCK* genes had FPKM values close to zero; seven genes exhibited relatively higher expression abundances; and others showed moderate expression abundances (Figure 11A). There were 13 *GhRLCK* genes whose expression levels were upregulated (Figure 11B) and 14 genes that were consistently downregulated at 6, 12, and 24 hpi (Figure 11C). Among them, the expression levels of nine genes were significantly changed during *V. dahliae infection*, with five genes (*GhRLCK49D*, *GhRLCK18A*, *GhRCLK7A*and *D*, and *GhRLCK43A*) highly indued and four genes (*GhRLCK22D*, *GhRLCK42D*, *GhRLCK9A*, and *GhRLCK64A*) notably depressed. The expression levels of these genes were then confirmed via RT-qPCR. Due to the high protein identity (98.83%) of *GhRLCK7A* and *D*(*GhRLCK7*), a pair of primers was used to amplify both genes at the same time. Eight genes exhibited expression patterns consistent with those in RNA seq. The expression levels of four downregulated genes (*GhRLCK22D*, *GhRLCK42D*, *GhRLCK9A*, and *GhRLCK64A*) were significantly reduced at 24 dpi, and four-fifths of the upregulated genes (*GhRLCK49D*, *GhRLCK18A*, and *GhRCLK7A*and *D*) were remarkably highly induced at both 6 hpi and 24 hpi, except *GhRLCK43A* (Figure 12).

### 2.8. Cis-Elements in GhRLCK Promoters

The 1500 bp upstream sequences of the initiation codons of *GhRLCKs* were employed for cis-element analysis. Many cis-elements were detected in the promoter regions of *GhRLCKs*. Here, the cis-acting elements related to hormone response and adversity response were counted and analyzed (Table S5). The plant hormone response elements mainly included the abscisic acid (ABA) response element (ABRE), salicylic acid response element (TCA-element) (GARE-motif, P-box, and TATC-box), methyl jasmonate response element (CGTCA-motif and TGACG-motif), ethylene response element (ERE), etc. [39]. Herein, 63 *GhRLCKs* had TCA-elements, 61 *GhRLCKs* had methyl jasmonate response elements (CGTCA-motif and TGACG-motif), 75 *GhRLCKs* had ABRE, 45 *GhRLCKs* had gibberellin response elements, and 96 *GhRLCKs* had ERE (Figure S3). The abiotic stress response elements mainly included the drought stress response element (MBS), low-temperature response element (LTR), defense and stress response elements (TC-rich repeats), and stress response elements (W box) [39]. Forty *GhRLCKs* had MBS, 37 *GhRLCKs* had LTR, 56 *GhRLCKs* had W box, and 35 *GhRLCKs* had TC-rich repeats (Figure S3). Therefore, the ERE element was remarkably rich in some *GhRLCKs*, for example, *GhRLCK28A*and *D*, *GhRLCK9A* and *D*, *GhRLCK47D,* and *GhRLCK10A*, and the ABA response element and methyl jasmonate response element were abundant in a portion of the *GhRLCKs* (Figure S3). Therefore, these genes may implicate the ethylene, methyl jasmonate, and ABA pathways in mediating cotton defense against abiotic and biotic stresses.

### 2.9. Silencing of GhRLCK7 Compromised Resistance to V. dahliae in Upland Cotton

**Figure 13:**
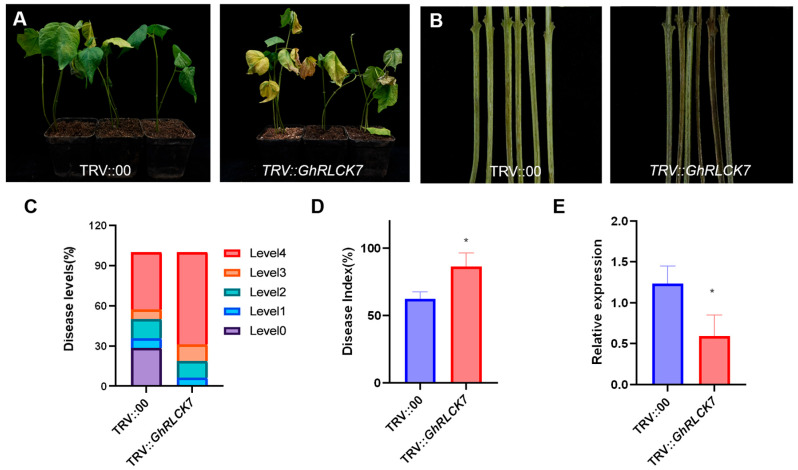
The silencing of *GhRLCK7* dampened upland cotton resistance to *V. dahliae*. (**A**) The leaf symptoms of TRV::00 and TRV:: *GhRLCK7* cotton plants after *V. dahliae* inoculation at 15 dpi. Two weeks after VIGS, the seedlings were inoculated with *V.dahliae* spores via the root dipping method. More than 15 TRV::00 or TRV:: *GhRLCK7* seedlings were included in individual assays. The experiments were repeated at least three times with similar results. (**B**) A comparison of vascular browning in the stems of TRV::00 and TRV::*GhRLCK7* plants at 15 dpi. (**C**) The disease levels at 15 dpi. (**D**) The disease index at 17 dpi. (**E**) The silencing efficiency of *GhRLCK7* in TRV::00 and TRV::*GhRLCK7* plants according to RT-qPCR. The data are presented as mean ± SE from three independent repeats. An asterisk represents significant differences compared with the results of TRV::00 according to two-tailed Student’s *t*-tests (* *p* < 0.05).

Based on the significant change in expression level upon *V. dahliae* infection, *GhRLCK7A* and *D* were selected to investigate their role in cotton’s response to *V. dahliae*. A VIGS vector (TRV::*GhRLCK7*) targeting both genes was constructed for gene silencing. After *V. dahliae* inoculation, *GhRLCK7*-silenced seedlings (TRV::*GhRLCK7*) exhibited more serious wilting, yellowing, and defoliation on their leaves, while the disease symptoms in the control (TRV::00) plants showed mild symptoms and only partial wilting was observed (Figure 13A). Meanwhile, the degree of vascular browning in TRV::*GhRLCK7* plants was much more severe than that in TRV::00 plants (Figure 13B). The severity of the disease was further recorded and evaluated by disease level and disease index. Fifteen days after *V. dahliae* inoculation, we documented the number of plants displaying each disease level (1–4). The ratios of TRV::*GhRLCK7* plants with their corresponding levels were as follows: 6.25%, level-1; 12.5%, level-2; 12.5%, level-3; and 68.75%, level-4, and those of the control plants were 28.57%, level-0; 7.14%, level-1; 14.29%, level-2; 7.14%, level-3; and 42.86%, level-4 (Figure 13C). The disease index of TRV::*GhRLCK7* plants (86.36%) was significantly higher than that of TRV::00 plants (62.28%) at 17 dpi (Figure 13D). Consistently, TRV::*GhRLCK7* plants were more susceptible to *V. dahliae* infection in terms of their disease symptoms, disease level, and disease index compared to TRV::00 plants. Meanwhile, the silencing efficiency of *GhRLCK7* was examined via RT-qPCR and the result showed that the expression level of *GhRLCK7* in TRV::*GhRLCK7* leaves was significantly reduced compared with that in TRV::00 leaves (Figure 13E), suggesting that *GhRLCK7* was successfully silenced in TRV::*GhRLCK7* plants. Collectively, the silencing of *GhRLCK7* makes cotton more susceptible to *V. dahliae* infection. 

## 3. Discussion

### 3.1. The Evolutionary Patterns of GhRLCKs

The RLCK-VII subfamily members are critical players in plant kinase-mediated signaling and the regulation of various cellular activities during plant growth, development, and defense against biotic and abiotic stresses [17], especially PAMP-triggered immunity signaling [20]. In this study, 129 members of the RLCK-VII subfamily were identified from the upland cotton genome (*G. hirsutum*, AD_1_, NAU assembly) through a genome-wide search and conserved domain identification referring to the homolog genes in *Arabidopsis*. The *GhRLCK* genes share a close evolutionary relationship with *Arabidopsis* RLCK-VII members and are divided into nine groups following the category of their *Arabidopsis* homologs. The gene structures and protein features of *GhRLCKs* are relatively conserved within a group. Therefore, *GhRLCK* members may exhibit similar functions to the *Arabidopsis* orthologs, and functional redundancy may exist among genes in the same group [20].

Genomic alterations, such as gene duplication and chromosomal rearrangements, have a substantial impact on the formation of gene families [40]. Tandem and segmental genome replication are two important factors in gene family expansion [41]. Through our analysis, 135 pairs of duplicated segment events and one tandem duplication event happened in *GhRLCK* gene expansions in upland cotton. Segmental duplication is the major mechanism contributing to the expansion of the *GhRLCK* subfamily. To explore the different selective constraints on the *GhRLCK* genes, the *Ka*/*Ks* ratios were computed for the duplicated genes. Although there were differences in the *Ka*/*Ks* values of the duplicated gene pairs, all the estimated *Ka*/*Ks* values were substantially less than one. Thus, the gene sequences of *GhRLCKs* within cotton underwent strong purifying selection pressures, and positive selections may have worked after tandem duplication and fragment duplication in this gene family throughout the evolution of cotton species [42]. This suggests that the cotton RLCK VII subfamily was relatively conserved throughout evolution.

### 3.2. Potential Functions of GhRLCK Genes in Cotton’s Response to Abiotic Stresses

The expression pattern of a gene in various tissues and organs of a plant could provide clues for the elucidation of its function and putatively involved pathways. *GhRLCKs* exhibit tissue-specific expression patterns, with variations in expression levels across different tissues, organs, and developmental stages. Several genes, including *GhRLCK50A*, *GhRLCK52A*and *D*, *GhRLCK70D*, and *GhRLCK46A*, exhibit specific expression in the roots, indicating that they may confer to root development, defense against soil-borne pathogens, or the absorption and utilization of nutrients from soil. *GhRLCK3D* exhibits specific expression in fiber and is barely expressed in other tissues, giving a hint that *GhRLCK3D* may play a role in cotton fiber elongation and development. *GhRLCK51A*and *D* exhibit a dominant expression pattern in the petal, anther, filament, and sepal, indicating their involvement in cotton flower development. Therefore, cotton *GhRLCKs* may play diverse roles in growth- and stress-related signaling pathways, as reported in other plants [17].

Consistent with our deduction, many *GhRLCKs* were found to be involved in responses to cold, heat, NaCl, and drought stress, whose expression levels were notably changed during stresses. Overall, 15 members were consistently upregulated and 17 members were suppressed during low-temperature treatment. In response to high temperature, drought, and NaCl stress, 27, 13, and 11 genes displayed increased transcript levels, and 17, 17, and 18 genes showed continuously decreased transcript levels, respectively. In addition, there were a few overlapping genes, for example, *GhRLCK49A*, *GhRLCK8A*, *GhRLCK24D*, *GhRLCK1A*, *GhRLCK4D*, *GhRLCK20A*, *GhRLCK54D*, *GhRLCK58D*, *GhRLCK66D*, and *GhRLCK23D*, which exhibited sustained upregulation or downregulation of expression among all these treatments, suggesting that they might be involved in cotton defense against cold, heat, NaCl, and drought stresses. A similar result has been reported on *OsGUDK*, an *RLCK-VII* member in rice. The expression of *OsGUDK* is induced by dehydration, NaCl, heat, and cold, and *OsGUDK* was demonstrated to regulate the response to salinity, drought, and ABA [30,31]. Moreover, more than three ABRE elements were found in the promoter of *GhRLCK58D*, *GhRLCK54D*, and *GhRLCK23D*, providing clues that these genes may participate in ABA signaling. An RLCK-VII member, *Esi47*, from wheatgrass has been reported to regulate salt stress and ABA signaling [32]. Taken together, *GhRLCK58D*, *GhRLCK54D*, and *GhRLCK23D* may act as the main regulatory genes in the ABA signaling pathway required for the abiotic stress response in cotton. 

### 3.3. Involvement of GhRLCKs in Cotton Defense against V. dahliae

Many RLCK VII genes play important roles in plant innate immunity [20]. Upon *V. dahliae* infection, many *GhRLCK* genes showed variations in their expression levels. Among them, 14 genes were consistently downregulated and 13 genes were consistently upregulated after *V. dahliae* infection. Five genes, including *GhRLCK49D*, *GhRLCK43A GhRLCK18A*, and *GhRCLK7A*and *D*, were highly induced, and another four genes, *GhRLCK22D*, *GhRLCK42D*, *GhRLCK9A*, and *GhRLCK64A*, were notably depressed. This result implies their involvement in cotton defense against *V. dahliae*. From the analysis of cis-elements in the promoter sequence, *GhRLCK7D* and *GhRLCK9A* had five and seven ERE elements in their promoters, suggesting that both genes may be involved in the response of cotton to ethylene. Ethylene is one of the most well-studied defense-related hormones [43] and the activation of the ethylene signaling pathway enhances the resistance of cotton to *V. dahliae* [44]. The expression of *GhRLCK7D* was highly induced, whereas the expression of *GhRLCK9A* was notably reduced, upon *V. dahliae* inoculation. Therefore, it is speculated that *GhRLCK7D* and *GhRLCK9A* may imply specific immune signaling other than ethylene during cotton defense against *V. dahliae*, which needs to be further explored.

*GhRLCK7A* and *D* are a pair of homologous genes and belong to the RLCK VII subfamily Group I. The Group I gene *AtPBL27* has been reported to mediate chitin-induced immune signaling relay and is essential for innate immunity in *Arabidopsis* [45,46]. A similar mechanism may be employed by *GhRLCK7*. The knock-down of *GhRLCK7* compromised cotton resistance against *V. dahliae*, which is underpinned by a severe disease phenotype on the leaves and stems, and an elevated disease index of *GhRLCK7*-silenced plants. Thus, *GhRLCK7* plays a positive role in defense against *V. dahliae* in cotton. The homolog of *GhRLCK7* in rice is *OsRLCK185*, which has been reported to function as a substrate of OsCERK1 to activate chitin-induced MAPK activation in rice [47,48]. Whether *GhRLCK7* confers to the chitin-induced immune response and shares the same characteristics as *OsRLCK185* in the chitin-triggered immunity of cotton is worth further study. 

## 4. Materials and Methods

### 4.1. Identification of RLCK-VII Subfamily Genes in Upland Cotton

The protein sequences of 46 RLCK VII subfamily genes in *A. thaliana* (Table S1) were obtained from the TAIR database (https:and/www.arabidopsis.org/ (accessed on 20 November 2022)) and used as query templates to search against the CottonFGD database (https://cottonfgd.net/ (accessed on 25 November 2022)) using BLASTp for the homologous genes in upland cotton. The cotton genome database used was *G. hirsutum* (AD_1_, NAU assembly). The genes with identities > 50% were selected from the retrieved list of each query, in which proteins were arranged in descending order of their bit scores. The genes obtained from all the queries were combined and the duplicate members were removed from the primary list. Next, the protein sequences of putative *GhRLCKs* were uploaded to the NCBI Conserved Domain Database (NCBI-CDD) to analyze the conserved domains (E-value = 1e^−2^). The ones that contained cytoplasmic kinase domains but not any extracellular domains or transmembrane motifs were confirmed to be *GhRLCKs*. SMART was used to further confirm the protein motifs and exclude genes that were not RLCKs [49]. 

### 4.2. Physicochemical Property Characterization of GhRLCK Proteins

The physicochemical properties of *GhRLCKs* in upland cotton were analyzed by uploading the protein sequences of each gene to Expasy ProtParam (http://web.expasy.org/protparam (accessed on 10 January 2023)), including the molecular weight (MW), instability index and fat index, and theoretical isoelectric point (pI).

### 4.3. Phylogenetic Analysis of GhRLCK Genes

The phylogenetic tree of RLCK-VII subfamily members in *G. hirsutum* and *A. thaliana* was constructed using TBtools v1.120 [50]. MUSCLE Wrapper was used for multiple sequence alignment, and then, TrimAL Wrapper was used to trim the results of the sequence alignment [51]. The phylogenetic tree was constructed using IQ-tree with the maximum likelihood (ML) method and ultrafast bootstrap with 5000 bootstrap replications [35]. Moreover, the phylogenetic tree was visualized and tidied using the online tool iTOL (https://itol.embl.de/ (accessed on 20 January 2023)).

### 4.4. Gene Structure and Conserved Domain Analysis

The conserved domains of RLCK-VII genes in *A. thaliana* and *G. hirsutum* were predicted using the Batch CD-search tool in the NCBI database using a threshold E-value of 1e^−2^ (https://www.ncbi.nlm.nih.gov/ (accessed on 18 December 2022)). The domain was displayed via concise results. The exon and intron structures of the genes were visualized using the gff3-file of *the G. hirsutum* genome structure annotation data. TBtools was used to merge and map the gene structure, conserved domain, and phylogenetic tree.

### 4.5. Chromosomal Location, Gene Duplication, and Synteny Analysis

The genome structure annotation file of upland cotton was downloaded from cotton FGD [52]. The location information of the gene on the chromosome was obtained from and visualized using the Gene Location Visualize module of TBtools. The synteny analysis of *GhRLCKs* in the *G. hirsutum* genome was conducted using MCScanX, and the tandem and segmental duplication events of homologous genes were analyzed based on the results of the synteny analysis [6]. In addition, a *Ka*/*Ks* calculator was used to calculate the ratio of the non-synonymous substitution rate (*Ka*) to the synonymous substitution rate (*Ks*) of *GhRLCK* gene pairs with duplication events [53]. 

### 4.6. Gene Expression Profile Analysis

The transcriptome datasets of the upland cotton variety “TM-1” in different tissues and organs (roots, stems, leaves, petals, anthers, bracts, filaments, pistils, sepals, torus, fibers, and ovules) and under different abiotic stresses (4 °C—cold, 37 °C—heat, salt, and drought) were downloaded from the cotton MD database [37]. The transcriptome dataset of TM-1 infected with the *V. dahliae* V991 isolate [38] was employed to analyze the expression changes of *GhRLCKs* in response to *V. dahliae* infection. The expression heatmaps of *GhRLCKs* were generated using TBtools. Fragments per kilobase of transcript per million mapped reads (FPKM) values were used to perform logarithmic normalization of the expression data.

### 4.7. Analysis of Promoter Regions for Cis-Elements

The 1500 bp DNA sequences upstream of the start codon of *GhRLCKs* were extracted using TBtools and submitted to the online database Plant CARE for the analysis of cis-elements [54].

### 4.8. Cultivation of Cotton and V. dahliae

*G. hirsutum* “Shanximian” was grown in a mixed matrix (soil: vermiculite = 2:1, *w*/*w*) in a greenhouse at 25 °C and 60% humidity with a 16 h light/8 h dark cycle. Two-week-old cotton plants were subjected to Agrobacterium-mediated VIGS assays. 

*V. dahliae* (V991 isolate) was grown on potato dextrose agar medium (PDA) at 25 °C for 4 days. The hyphae of V991 were inoculated into potato dextrose broth medium (PDB) and cultured at 25 °C with shaking (200 rpm) for 6 days. The supernatant of the culture was filtered with four-layer sterile gauze to produce a spore suspension. The spore suspension of V991 was adjusted at a concentration of 1 ×10^6^ conidia/mL with sterile water for inoculation. The root dipping method was employed for *V. dahliae* inoculation on cotton seedlings as described previously [33].

### 4.9. Treatments of Cotton with Abiotic and Biotic Stresses

Cotton seeds were germinated in a plastic box containing wet filter papers at room temperature for three days. The germinated seedlings were transplanted in sterile water. Five days after transplantation, the seedlings were subjected to cold (4 °C), heat (37 °C), 20% PEG, and 200 mM NaCl treatments, and leaf samples were collected at indicated time points, respectively. The *V. dahliae* treatment was performed in an inoculum of 1 × 10^8^ conidia/mL suspension using the root dipping method and the roots were harvested at 0, 6, and 24 h after treatment. The treatments were performed with three replicates. The samples were stored at −80 °C for RNA isolation.

### 4.10. RNA Extraction and Real-Time Quantitative PCR (RT-qPCR)

Total RNA was obtained from the cotton samples using an RNA extraction kit (Biomed Gene Technology, Co., Ltd., Beijing, China). The cDNA was synthesized using a PrimeScriptTM RT reagent Kit with gDNA Eraser (Perfect Real Time) (TaKaRa Bio, Dalian, China). RT-qPCR was performed using SYBR Premix Ex Taq (Tli Rnase H Plus) (TaKaRa) on an ABI 7500 thermocycler (Applied Biosystems, Foster City, CA, USA). GhUBQ7 (DQ116441) was utilized as an internal standard gene in cotton. The 2^−ΔΔCT^ method was used to determine the relative expression levels of the genes. The primers used are shown in Table S6.

### 4.11. Construction of VIGS Vector and Implementation of VIGS

The binary TRV vectors pTRV-RNA1 and pTRV-RNA2 (pYL56) were used for VIGS in cotton [55]. The 470 bp of the *GhRLCK7* fragment was amplified from cotton cDNA, and then, inserted into the pYL156 vector via the *EcoR* I/*Kpn* I enzyme sites. The recombinant plasmid of pYL156-*GhRLCK7* was verified via sequencing and transformed into *A. tumefaciens* (strain GV3101). 

The *Agrobacterium* strains containing pTRV-RNA1, pYL156-*GhCLA1*, or pYL156-*GFP* (control) plasmids were stocked in our laboratory [33] and cultured together with pTRV-*GhRLCK7 Agrobacterium* for VIGS infiltration. The implementation of VIGS was conducted on two-week-old cotton seedlings following the instructions reported previously [55]. The plant albino phenotype resulting from the silencing of *GhCLA1* was used as a visual marker to indicate the successful silencing of *GhCLA1*. Two weeks after VIGS, when the photobleaching phenotype was observed on the new leaves of *GhCLA1*-silenced cotton seedlings, *GhRLCK7-*silenced seedlings and control seedlings were subjected to *V. dahliae* challenge.

### 4.12. Disease Evaluation

The disease severity of cotton seedlings was evaluated via leaf symptoms, stem discoloration, and disease index.

The DI formula

(1) $$(1n_{1}+2n_{2}+3n_{3}+4n_{4})\times 100/4N_{t}$$

was used to calculate the disease index, where *n*_1_ to *n*_4_ represent the number of plants in each category and *N_t_* represents the total number of plants tested. The disease symptoms of cotton seedlings subjected to VIGS were recorded using a 0-to-4 rating scale as reported in [33]. Briefly, 0 indicates no visible chlorosis or wilting symptoms; 1 represents one true leaf showing chlorosis or wilting symptoms; 2 indicates that two true leaves have wilted or dropped off; 3 means that more than two true leaves have wilted or dropped off; and the whole plant wilting or all leaves dropping off corresponds to a value of 4. 

## 5. Conclusions

This study explored the composition, evolution relationship, and function analysis of RLCK-VII subfamily genes in upland cotton via genome-wide identification, phylogeny, duplication events, expression patterns, and VIGS. *GhRLCKs* is a large group with 129 members, and its genes undergo purifying selection driven by segmental duplication and tandem duplication. Segmental duplication plays the dominant role during the expansion of this gene family. In this paper, diverse expression patterns of *GhRLCKs* are revealed in response to abiotic stresses and *V. dahliae* infection. Several genes that implicate more than one stressor are speculated to rely on ABA signaling. *GhRLCK7* is demonstrated to be a positive regulator in cotton’s defense against *V. dahliae* infection. Our results provide insights into the essential details of RLCKs and lay the foundation for in-depth functional analysis of the RLCK-VII subfamily genes in upland cotton.

## Figures and Tables

**Table 1:** Basic information about *Gossypium hirsutum* RLCK V-II family members.

Gene Locus ID	Name	Length/bp	CDS Length/bp	Number of Exons	Number of Amino Acids	Molecular Weight /Da	Theoretical pI	Instability Index	Aliphatic Index
Gh_A01G0195	GhRLCK8A	3040	1554	5	517	57,041.98	9.09	37.98	66.63
Gh_A01G0607	GhRLCK1A	1912	1176	5	391	43,474.88	4.95	40.14	75.37
Gh_A01G0842	GhRLCK22A	3180	1182	6	393	43,634.61	8.83	37.12	82.62
Gh_A01G1911	GhRLCK63A	2752	1116	6	371	41,226.76	9.25	42.22	79.92
Gh_A01G2145	GhRLCK11A	3657	1200	5	399	44,214.81	9.53	34.11	75.81
Gh_A02G0005	GhRLCK45A	3462	1281	6	426	47,181.73	8.91	35.75	83.10
Gh_A02G0870	GhRLCK59A	3266	1272	6	423	46,399.66	9.29	26.04	75.15
Gh_A02G1028	GhRLCK68A	1947	1437	6	478	52,031.13	6.56	55.10	86.67
Gh_A02G1701	GhRLCK58A	7322	1275	6	424	47,405.54	8.79	36.09	74.95
Gh_A03G0052	GhRLCK47A	2196	1002	5	333	36,755.70	9.25	42.84	77.93
Gh_A03G0179	GhRLCK46A	5702	1197	7	398	44,844.30	6.28	38.58	87.71
Gh_A03G1241	GhRLCK35A	6991	1047	7	348	39,554.32	9.03	43.49	85.43
Gh_A03G1511	GhRLCK55A	2608	1266	7	421	47,057.59	9.67	36.11	77.58
Gh_A03G2069	GhRLCK61A	1906	1287	6	428	47,415.63	9.19	40.87	80.02
Gh_A04G0613	GhRLCK31A	2600	1224	4	407	46,151.73	9.60	41.83	79.48
Gh_A04G1161	GhRLCK33A	3355	1080	7	359	40,469.48	9.00	44.45	83.90
Gh_A05G0076	GhRLCK40A	1683	1305	6	434	48,511.47	9.51	38.21	83.57
Gh_A05G0253	GhRLCK53A	1709	1356	5	451	51,229.67	9.17	41.72	79.33
Gh_A05G0599	GhRLCK66A	2513	1149	6	382	41,979.78	9.44	29.68	79.42
Gh_A05G0809	GhRLCK28A	1940	1401	4	466	52,446.62	8.75	36.72	83.43
Gh_A05G0810	GhRLCK60A	2316	1218	5	405	44,786.69	9.58	36.34	74.96
Gh_A05G1164	GhRLCK48A	1729	1344	5	477	50,704.40	9.43	43.57	85.46
Gh_A05G2537	GhRLCK29A	2178	1281	4	426	47,965.80	9.49	43.98	84.44
Gh_A05G2570	GhRLCK10A	3045	1152	5	383	42,251.36	9.26	27.27	81.78
Gh_A05G3044	GhRLCK64A	2475	1236	6	411	45,474.55	9.45	42.55	76.72
Gh_A05G3371	GhRLCK19A	2613	1557	5	518	57,795.10	7.61	58.61	61.18
Gh_A05G3532	GhRLCK42A	4149	1185	5	394	44,234.99	6.35	36.28	76.24
Gh_A06G0688	GhRLCK54A	1610	1332	4	443	49,645.87	8.98	44.06	80.56
Gh_A06G0724	GhRLCK50A	1621	1017	5	338	38,136.74	8.03	30.61	85.38
Gh_A06G1294	GhRLCK4A	1817	1170	6	389	43,541.75	6.12	35.59	85.73
Gh_A07G0319	GhRLCK41A	2954	1284	6	427	47,613.07	9.23	32.50	78.99
Gh_A07G2351	GhRLCK51A	1974	1152	7	383	43,458.78	7.67	42.42	85.74
Gh_A08G0138	GhRLCK44A	5083	1356	6	454	50,793.98	9.22	37.85	79.19
Gh_A08G0245	GhRLCK26A	2441	1251	4	416	46,582.46	9.09	36.01	82.96
Gh_A08G1460	GhRLCK23A	1862	1170	4	389	43,793.83	9.51	40.09	76.89
Gh_A08G2568	GhRLCK14A	1875	1269	5	422	46,882.09	8.04	36.85	75.12
Gh_A09G0212	GhRLCK36A	3028	1140	6	379	42,155.86	9.35	52.32	68.97
Gh_A09G0371	GhRLCK21A	3221	1284	6	427	47,536.09	8.28	25.85	77.24
Gh_A09G0599	GhRLCK6A	4986	1545	5	514	56,646.53	9.10	34.06	66.42
Gh_A09G1001	GhRLCK67A	3885	1236	6	411	45,302.56	9.30	26.20	77.59
Gh_A09G1391	GhRLCK32A	2426	1323	4	440	49,995.39	9.47	47.08	83.50
Gh_A09G1456	GhRLCK62A	1787	1194	6	397	44,277.17	9.78	39.91	76.17
Gh_A09G2123	GhRLCK5A	2527	1506	5	501	55,546.36	8.80	36.21	68.94
Gh_A10G0498	GhRLCK49A	2140	1404	6	467	52,688.41	9.33	39.45	88.09
Gh_A10G1428	GhRLCK7A	4007	1548	5	515	56,653.56	8.99	38.89	68.02
Gh_A10G1831	GhRLCK30A	2502	1290	4	429	49,065.08	9.71	42.53	82.03
Gh_A10G1861	GhRLCK9A	2450	1152	5	323	35,908.23	8.68	31.52	89.72
Gh_A10G1973	GhRLCK65A	2506	1161	6	386	42,796.62	9.12	35.70	76.55
Gh_A11G0259	GhRLCK25A	1764	1164	4	387	43,458.52	9.65	35.03	78.32
Gh_A11G0581	GhRLCK20A	2382	1242	5	413	45,673.01	8.48	34.63	73.22
Gh_A11G0673	GhRLCK27A	2199	1317	4	438	49,421.60	9.35	39.55	79.50
Gh_A11G1264	GhRLCK16A	1666	1290	5	429	47,253.97	5.81	37.23	73.52
Gh_A11G2714	GhRLCK17A	3936	1710	5	569	61,686.29	6.08	48.05	62.46
Gh_A11G3042	GhRLCK37A	3132	1431	6	476	52,748.79	9.13	48.41	71.89
Gh_A12G0114	GhRLCK57A	2037	1200	6	399	43,784.99	9.15	36.77	78.95
Gh_A12G1525	GhRLCK69A	3644	1266	6	421	46,018.49	9.73	41.96	77.41
Gh_A12G1932	GhRLCK24A	2259	1170	5	389	43,452.70	9.67	38.02	81.18
Gh_A13G0455	GhRLCK38A	3979	1461	6	486	54,009.00	9.09	39.93	71.46
Gh_A13G0572	GhRLCK56A	2997	1233	6	410	45,260.38	9.69	36.86	77.80
Gh_A13G0635	GhRLCK70A	2995	1263	6	420	45,829.34	9.54	39.99	79.19
Gh_A13G0755	GhRLCK18A	2701	1281	5	426	47,179.57	6.30	42.63	79.27
Gh_A13G1685	GhRLCK52A	1680	987	5	328	36,794.49	6.63	44.02	71.98
Gh_A13G1811	GhRLCK43A	5138	1323	6	440	49,149.66	6.58	36.73	72.73
Gh_A13G2001	GhRLCK15A	2461	1260	5	419	46,393.73	8.02	24.60	77.28
Gh_D01G0203	GhRLCK13D	4288	1392	5	463	50,970.19	6.52	35.26	71.86
Gh_D01G0204	GhRLCK12D	4545	1392	5	463	51,216.57	7.63	35.17	73.11
Gh_D01G0620	GhRLCK1D	1877	1161	5	386	42,940.31	4.99	40.17	74.82
Gh_D01G0869	GhRLCK22D	3258	1146	5	381	42,271.09	8.44	37.20	82.91
Gh_D01G1270	GhRLCK35D	1882	1077	7	358	40,378.33	9.12	39.34	83.83
Gh_D01G2115	GhRLCK11D	3739	1200	5	399	44,211.89	9.48	32.54	78.02
Gh_D01G2170	GhRLCK63D	2700	1116	6	372	41,389.03	9.54	41.45	79.65
Gh_D01G2270	GhRLCK8D	3044	1554	5	517	56,787.69	9.02	37.16	66.63
Gh_D02G0019	GhRLCK45D	3446	1281	6	426	47,210.73	8.91	35.55	82.39
Gh_D02G1680	GhRLCK36D	2136	1128	7	375	42,423.62	8.89	45.91	85.52
Gh_D02G1980	GhRLCK55D	2018	1233	6	410	45,604.95	9.53	34.19	76.80
Gh_D03G0018	GhRLCK58D	2263	1251	6	416	46,384.36	8.81	33.93	74.04
Gh_D03G0702	GhRLCK68D	1942	1434	6	477	51,918.05	8.06	52.13	89.08
Gh_D03G1405	GhRLCK46D	7154	1200	7	399	44,896.47	6.61	37.33	88.72
Gh_D03G1604	GhRLCK47D	2179	1338	5	445	49,394.48	9.61	36.70	81.30
Gh_D04G0075	GhRLCK43D	4213	1185	5	394	44,283.03	6.35	35.79	75.25
Gh_D04G0266	GhRLCK19D	1221	1128	2	375	42,145.62	8.20	63.01	60.08
Gh_D04G0458	GhRLCK40D	3425	1479	6	492	54,423.71	9.35	42.40	69.59
Gh_D04G1073	GhRLCK32D	3150	1368	5	455	51,269.75	9.64	41.39	79.89
Gh_D04G1772	GhRLCK34D	3351	1080	7	359	40,453.42	9.05	42.48	83.62
Gh_D05G0134	GhRLCK41D	1700	1290	6	369	41,304.47	9.59	36.56	88.48
Gh_D05G0345	GhRLCK53D	1704	1356	5	451	51,241.70	9.07	40.22	78.47
Gh_D05G0730	GhRLCK66D	2424	1149	6	382	41,999.77	9.45	28.69	78.93
Gh_D05G0928	GhRLCK28D	1396	990	4	329	37,551.18	8.28	34.95	93.62
Gh_D05G0929	GhRLCK60D	2365	1218	6	405	44,664.62	9.53	37.32	75.46
Gh_D05G1341	GhRLCK48D	1728	1344	5	447	50,614.22	3.39	44.57	87.20
Gh_D05G2814	GhRLCK30D	2122	1284	4	427	48,170.15	9.55	44.13	83.77
Gh_D05G2857	GhRLCK10D	3012	1152	5	383	42,268.30	9.20	27.68	81.78
Gh_D06G0789	GhRLCK54D	1610	1332	4	443	49,756.95	9.05	42.82	79.46
Gh_D06G0842	GhRLCK50D	1887	1296	5	431	48,285.41	7.57	30.46	89.33
Gh_D06G1617	GhRLCK4D	1617	1167	5	388	43,262.31	5.72	37.23	86.21
Gh_D07G0159	GhRLCK51D	1791	1347	4	448	50,522.14	8.55	33.40	88.75
Gh_D07G0376	GhRLCK42D	3004	1284	6	427	47,648.11	9.29	33.12	78.99
Gh_D08G0016	GhRLCK14D	1854	1269	5	422	46,941.23	8.36	34.34	75.12
Gh_D08G1756	GhRLCK23D	1869	1170	4	389	43,814.90	9.49	43.00	80.15
Gh_D09G0202	GhRLCK37D	3015	1479	5	480	53,840.51	9.54	46.34	75.38
Gh_D09G0393	GhRLCK21D	3290	1284	6	427	47,548.21	8.25	25.63	78.13
Gh_D09G0598	GhRLCK6D	5006	1545	5	514	56,753.74	9.20	34.63	67.20
Gh_D09G1021	GhRLCK67D	3802	1236	6	411	45,326.54	9.30	26.71	76.42
Gh_D09G1396	GhRLCK33D	2382	1299	4	432	49,364.69	9.52	41.66	80.97
Gh_D09G2328	GhRLCK5D	2534	1506	5	501	55,342.20	8.90	37.84	70.70
Gh_D10G0142	GhRLCK3D	3818	1209	6	402	44,636.94	6.77	27.78	93.38
Gh_D10G0523	GhRLCK49D	1677	1368	5	455	51,016.70	9.17	35.66	90.86
Gh_D10G1669	GhRLCK7D	4019	1548	5	515	56,700.66	9.07	37.10	68.21
Gh_D10G2126	GhRLCK9D	2466	1152	5	383	42,414.34	8.96	28.25	80.26
Gh_D10G2531	GhRLCK31D	2505	1290	4	429	48,987.01	9.71	42.53	82.73
Gh_D11G0278	GhRLCK25D	1816	1164	4	387	43,406.49	9.65	34.94	79.33
Gh_D11G0666	GhRLCK20D	2669	1242	5	413	45,792.30	8.96	32.93	74.41
Gh_D11G0732	GhRLCK38D	3096	1431	6	476	52,729.83	9.26	47.90	71.28
Gh_D11G0788	GhRLCK27D	2199	1317	4	438	49,406.51	9.37	40.98	78.84
Gh_D11G1413	GhRLCK16D	1667	1290	5	429	47,135.87	5.81	36.45	74.66
Gh_D11G2878	GhRLCK2D	1816	1134	5	377	42,497.91	8.43	39.47	92.28
Gh_D11G3068	GhRLCK17D	3797	1677	5	558	60,534.28	6.85	45.87	63.51
Gh_D12G0126	GhRLCK57D	2040	1200	6	399	43,852.03	9.15	38.26	79.67
Gh_D12G1647	GhRLCK69D	3772	1266	6	421	46,004.51	9.76	42.23	76.72
Gh_D12G2113	GhRLCK24D	1859	1161	4	386	43,117.45	9.73	40.05	81.32
Gh_D13G0556	GhRLCK56D	2958	1233	6	410	45,274.36	9.56	36.25	79.00
Gh_D13G0697	GhRLCK39D	3979	1461	6	486	53,902.97	9.20	40.46	71.67
Gh_D13G0752	GhRLCK70D	2944	1263	6	420	45,827.33	9.54	40.94	78.50
Gh_D13G0942	GhRLCK18D	2743	1281	5	426	47,120.45	6.00	42.66	78.83
Gh_D13G2164	GhRLCK44D	5149	1281	6	426	47,696.18	8.40	37.30	73.97
Gh_D13G2375	GhRLCK29D	2178	1422	4	473	53,422.69	8.06	34.63	81.59
Gh_D13G2376	GhRLCK61D	2179	1209	6	402	44,454.33	9.43	33.59	76.89
Gh_D13G2400	GhRLCK15D	2466	1260	5	419	46,465.84	8.48	23.98	77.04
Gh_D13G2490	GhRLCK52D	1737	1383	5	460	51,581.21	6.66	46.09	74.24

## Data Availability

The protein sequences of the 46 RLCK VII subfamily genes in *A.thaliana* were obtained from the TAIR database and used as query templates to search against the CottonFGD database (https://cottonfgd.net/ (accessed on 25 November 2022)) using BLASTp. The raw data of RNA-seq were downloaded from the cotton MD database (https://yanglab.hzau.edu.cn/CottonMD) (accessed on 15 June 2023).

## Supplementary Materials

The following supporting information can be downloaded at: https://www.mdpi.com/article/10.3390/plants12173170/s1, Figure S1: Distribution of *GhRLCKs* on chromosomes. Figure S2: Expression levels of *GhRLCKs* in different tissues/organs of upland cotton. Figure S3: The cis-regulatory elements in *GhRLCK* promoter regions. Table S1: The groupings of the 46 *RLCK-VII* subfamily members in *Arabidopsis thaliana*. Table S2: The grouping of the 129 *RLCK-VII* subfamily members in upland cotton. Table S3: The gene pairs detected via synteny analysis. Table S4: Ka/Ks calculation of the duplicated RLCK V-II genes in *Gossypium hirsutum.* Table S5: The cis-elements in the promoter of *GhRLCKs.* Table S6: Primers used in this study.
