# Supplementary material for: Genome-Wide Identification and Expression Analysis of RLCK-VII Subfamily Genes Reveal Their Roles in Stress Responses of Upland Cotton
Source: Plants (Basel). 2023 Sep 4;12(17):3170. doi: 10.3390/plants12173170 (PMC10490013; doi:10.3390/plants12173170)
Supplement: Supplementary file 1 [file plants-12-03170-s001.zip › plants-2533066-supplementary.pdf]

The genome-wde identification and expression analysis of RLCK-VII subfamily genes reveal their roles in stress responses of upland cotton

Yuhan Cen[1,2+], Shiyi Geng[1,2+], Linying Gao[1], Xinyue Wang[1,2], Xin Yan[1], Yuxia Hou[1], Ping Wang[1, 2*]

[1] Pesticide Research, Department of Applied Chemistry, College of Science, China Agricultural University, Beijing 100193, China

[2] Key Laboratory of National Forestry and Grassland Administration on Pest Chemical Control, China Agricultural University, Beijing 100193, China

+These authors contributed equally to this work.

* Correspondence: wpingcau@163.com； Tel. 00-86-010-62733824

Supplemental materials

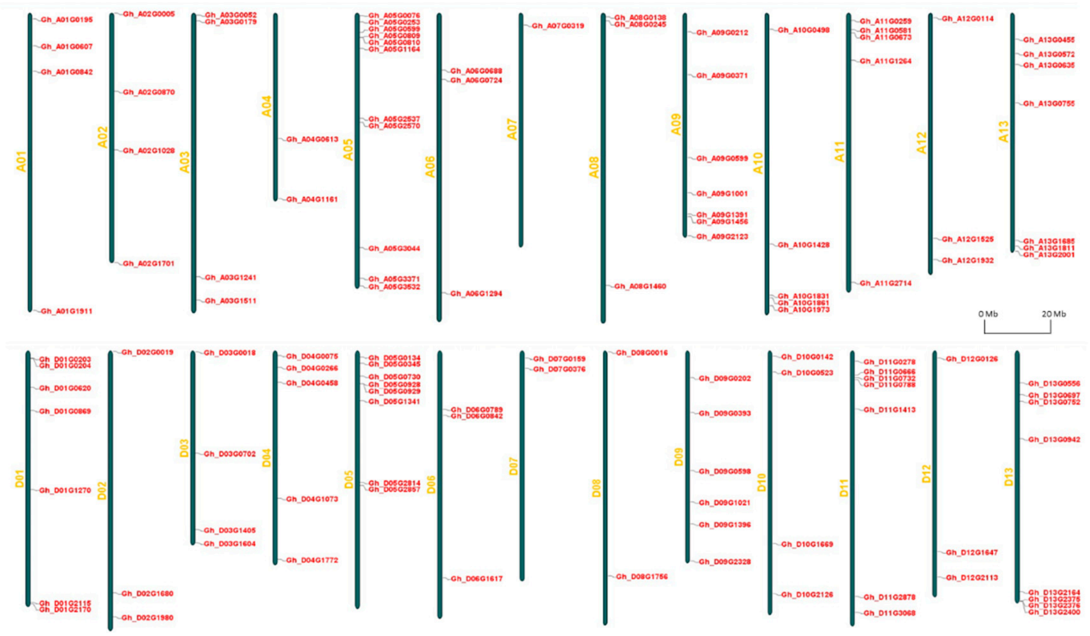

Figure S1. Distribution of *GhRLCKs* on chromosomes. *GhRLCKs* on the At subgenome of *G. hirsutum* (Top panel). *GhRLCKs* on Dt subgenome of *G. hirsutum* (Lower panel).

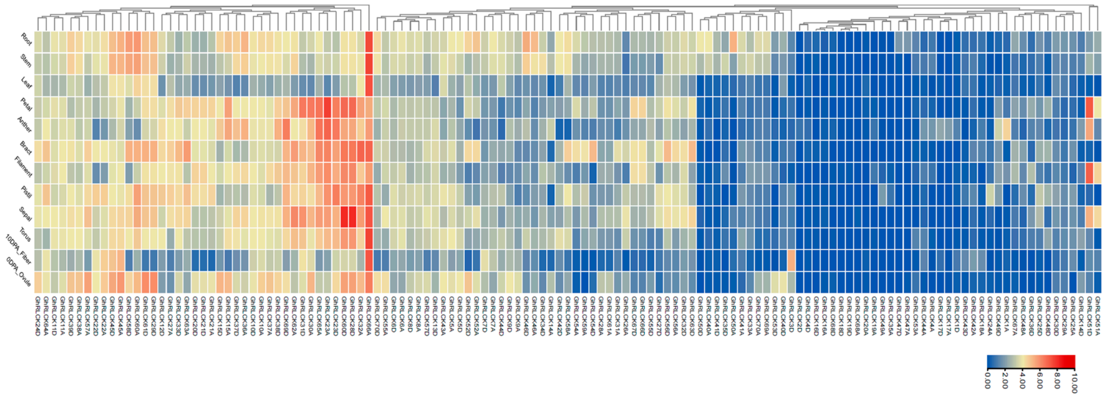

Figure S2. Transcriptional profiling of *GhRLCKs* in upland cotton different tissues/organs. Heat map showing the expression level of the 129 *GhRLCKs* genes in various tissues (root, stem, leaf, petal, anther, bract, filament, pistil, sepal, torus, 10 DPA fiber, and 0 DPA ovule) of TM-1 based on RNA-seq data. The scale bar is presented underneath the charts.

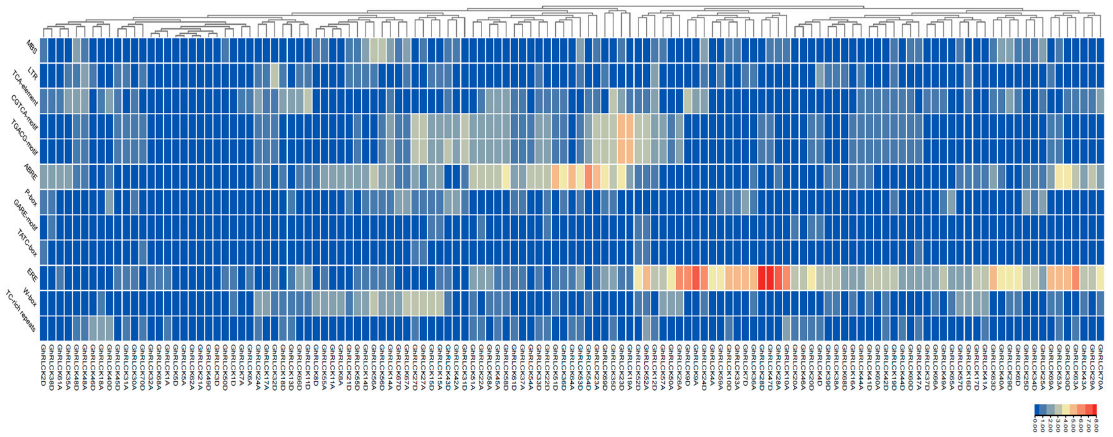

Figure S3. The cis-regulatory elements in *GhRLCKs* promoter regions. MBS, drought stress response element; LTR, low-temperature responsiveness element; TCA-element, salicylic acid responsiveness element; CGTCA-motif, MeJA-responsiveness cis-acting regulatory element; TGACG-motif, MeJA responsiveness element; ABRE, abscisic acid responsiveness element; P-box, GARE-motif; TATC-box, gibberellin-responsive element; TC-rich repeats, defense and stress responsiveness cis-element; W-box, plant defense signaling cis-acting element; ERE: ethylene response element; The colors indicate the number of cis-acting elements in *GhRLCK* genes. The scale bar is presented underneath the charts.

Table S1. The groupings of the 46 *RLCK-VII* subfamily members in *A. thaliana*

ID	Gene Name	ID	Gene Name	ID	Gene Name
**Group Ⅰ**		**Group Ⅳ**		**Group Ⅶ**	
AT2G28590	AtPBL6	AT1G24030	AtPBL28	AT1G74490	AtPBL29
AT1G07870	AtPBL5	AT4G17660	AtPBL20	AT1G26970	AtPBL4
AT5G02800	AtPBL7	AT5G47070	AtPBL19	AT1G69790	AtPBL18
AT5G18610	AtPBL27	AT2G28940	AtPBL37	AT1G14370	AtPBL2
AT5G13160	AtPBS1	AT2G39110	AtPBL38	AT2G02800	AtPBL3
AT1G61860	AtPBL41	AT5G03320	AtPBL40	**Group Ⅷ**	
AT3G20530	AtPBL23	AT3G09830	AtPBL39	AT3G28690	AtPBL36
**Group Ⅱ**		**Group Ⅴ**		AT5G15080	AtPBL34
AT3G26940	AtCDG1	AT4G35600	AtPBL30	AT3G01300	AtPBL35
AT1G76370	AtPBL22	AT2G17220	AtPBL32	**Group Ⅸ**	
AT1G20650	AtPBL21	AT1G76360	AtPBL31	AT1G61590	AtPBL15
**Group Ⅲ**		**Group Ⅵ**		AT1G72540	AtPBL33
AT5G16500	AtPBL43	AT3G55450	AtPBL1	AT2G26290	AtPBL12
AT3G02810	AtPBL42	AT2G39660	AtBIK1	AT5G35580	AtPBL13
AT4G13190	AtPBL24	AT2G28930	AtPBL10	AT2G05940	AtPBL14
AT3G07070	AtPBL26	AT1G07570	AtPBL9	AT5G56460	AtPBL16
AT3G24790	AtPBL25	AT5G02290	AtPBL11	AT5G01020	AtPBL8
				AT2G07180	AtPBL17

Table S2. The grouping of the 129 *RLCK-VII* subfamily members in upland cotton

ID	Gene Name	ID	Gene Name	ID	Gene Name
**Group Ⅰ**		Gh_D01G1270	*GhRLCK35D*	Gh_A13G0635	*GhRLCK70A*
Gh_D08G0016	*GhRLCK14D*	Gh_D04G1772	*GhRLCK34D*	Gh_D13G0752	*GhRLCK70D*
Gh_A08G2568	*GhRLCK14A*	Gh_A04G1161	*GhRLCK33A*	Gh_D12G1647	*GhRLCK69D*
Gh_D13G2400	*GhRLCK15D*	Gh_D11G0278	*GhRLCK25D*	Gh_A12G1525	*GhRLCK69A*
Gh_A13G2001	*GhRLCK15A*	Gh_A11G0259	*GhRLCK25A*	Gh_A09G1001	*GhRLCK67A*
Gh_D01G2115	*GhRLCK11D*	Gh_D12G2113	*GhRLCK24D*	Gh_D09G1021	*GhRLCK67D*
Gh_A01G2145	*GhRLCK11A*	Gh_A12G1932	*GhRLCK24A*	Gh_A05G0599	*GhRLCK66A*
Gh_A05G2570	*GhRLCK10A*	Gh_A08G1460	*GhRLCK23A*	Gh_D05G0730	*GhRLCK66D*
Gh_D05G2857	*GhRLCK10D*	Gh_D08G1756	*GhRLCK23D*	**GroupⅧ**	
Gh_A10G1861	*GhRLCK9A*	Gh_A08G0245	*GhRLCK26A*	Gh_D07G0376	*GhRLCK42D*
Gh_D10G2126	*GhRLCK9D*	Gh_A11G0673	*GhRLCK27A*	Gh_A07G0319	*GhRLCK41A*
Gh_D09G2328	*GhRLCK5D*	Gh_D11G0788	*GhRLCK27D*	Gh_A05G0076	*GhRLCK40A*
Gh_A09G2123	*GhRLCK5A*	Gh_D13G2375	*GhRLCK29D*	Gh_D05G0134	*GhRLCK41D*
Gh_D09G0598	*GhRLCK6D*	Gh_D05G0928	*GhRLCK28D*	Gh_D09G0202	*GhRLCK37D*
Gh_A09G0599	*GhRLCK6A*	Gh_A05G0809	*GhRLCK28A*	Gh_A09G0212	*GhRLCK36A*
Gh_A01G0195	*GhRLCK8A*	Gh_A10G1831	*GhRLCK30A*	Gh_D11G0732	*GhRLCK38D*
Gh_D01G2270	*GhRLCK8D*	Gh_D10G2531	*GhRLCK31D*	Gh_A11G3042	*GhRLCK37A*
Gh_D10G1669	*GhRLCK7D*	Gh_A05G2537	*GhRLCK29A*	Gh_D04G0458	*GhRLCK40D*
Gh_A10G1428	*GhRLCK7A*	Gh_D05G2814	*GhRLCK30D*	Gh_A13G0455	*GhRLCK38A*
Gh_D01G0203	*GhRLCK13D*	Gh_D04G1073	*GhRLCK32D*	Gh_D13G0697	*GhRLCK39D*
Gh_D01G0204	*GhRLCK12D*	Gh_A04G0613	*GhRLCK31A*	**Group Ⅸ**	
Gh_D10G0142	*GhRLCK3D*	Gh_A09G1391	*GhRLCK32A*	Gh_A03G0052	*GhRLCK47A*
Gh_A06G1294	*GhRLCK4A*	Gh_D09G1396	*GhRLCK33D*	Gh_D03G1604	*GhRLCK47D*
Gh_D06G1617	*GhRLCK4D*	**Group Ⅴ**		Gh_D05G1341	*GhRLCK48D*
Gh_D11G2878	*GhRLCK2D*	Gh_D03G0018	*GhRLCK58D*	Gh_A05G1164	*GhRLCK48A*
Gh_D01G0620	*GhRLCK1D*	Gh_A02G1701	*GhRLCK58A*	Gh_A10G0498	*GhRLCK49A*
Gh_A01G0607	*GhRLCK1A*	Gh_A12G0114	*GhRLCK57A*	Gh_D10G0523	*GhRLCK49D*
**Group Ⅱ**		Gh_D12G0126	*GhRLCK57D*	Gh_A06G0724	*GhRLCK50A*
Gh_A09G0371	*GhRLCK21A*	Gh_A13G0572	*GhRLCK56A*	Gh_D06G0842	*GhRLCK50D*
Gh_D09G0393	*GhRLCK21D*	Gh_D13G0556	*GhRLCK56D*	Gh_A07G2351	*GhRLCK51A*
Gh_D11G0666	*GhRLCK20D*	Gh_D02G1980	*GhRLCK55D*	Gh_D07G0159	*GhRLCK51D*
Gh_A11G0581	*GhRLCK20A*	Gh_A03G1511	*GhRLCK55A*	Gh_A13G1685	*GhRLCK52A*
Gh_A01G0842	*GhRLCK22A*	Gh_A02G0870	*GhRLCK59A*	Gh_D13G2490	*GhRLCK52D*
Gh_D01G0869	*GhRLCK22D*	**Group Ⅵ**		Gh_D06G0789	*GhRLCK54D*
**Group Ⅲ**		Gh_A05G3044	*GhRLCK64A*	Gh_A06G0688	*GhRLCK54A*
Gh_D11G3068	*GhRLCK17D*	Gh_A10G1973	*GhRLCK65A*	Gh_A05G0253	*GhRLCK53A*
Gh_A11G2714	*GhRLCK17A*	Gh_A03G2069	*GhRLCK61A*	Gh_D05G0345	*GhRLCK53D*
Gh_D13G0942	*GhRLCK18D*	Gh_D05G0929	*GhRLCK60D*	Gh_A03G0179	*GhRLCK46A*
Gh_A13G0755	*GhRLCK18A*	Gh_A05G0810	*GhRLCK60A*	Gh_D03G1405	*GhRLCK46D*
Gh_A05G3371	*GhRLCK19A*	Gh_D13G2376	*GhRLCK61D*	Gh_D02G0019	*GhRLCK45D*
Gh_D04G0266	*GhRLCK19D*	Gh_A09G1456	*GhRLCK62A*	Gh_A02G0005	*GhRLCK45A*
Gh_A11G1264	*GhRLCK16A*	Gh_D01G2170	*GhRLCK63D*		

Gh_D11G1413	*GhRLCK16D*	Gh_A01G1911	*GhRLCK63A*
**Group Ⅳ**		**Group Ⅶ**	
Gh_A03G1241	*GhRLCK35A*	Gh_A02G1028	*GhRLCK68A*
Gh_D02G1680	*GhRLCK36D*	Gh_D03G0702	*GhRLCK68D*

Table S3. The gene pairs detected by synteny analysis

Gene 1	Gene 2	Gene 1	Gene 2	Gene 1	Gene 2
GhRLCK8A	*GhRLCK6A*	*GhRLCK54A*	*GhRLCK51D*	*GhRLCK69A*	*GhRLCK70A*
GhRLCK8A	*GhRLCK7A*	*GhRLCK54A*	*GhRLCK49D*	*GhRLCK69A*	*GhRLCK70D*
GhRLCK8A	*GhRLCK6D*	*GhRLCK50A*	*GhRLCK53D*	*GhRLCK24A*	*GhRLCK23D*
GhRLCK8A	*GhRLCK7D*	*GhRLCK4A*	*GhRLCK1D*	*GhRLCK24A*	*GhRLCK27D*
GhRLCK1A	*GhRLCK4A*	*GhRLCK4A*	*GhRLCK3D*	*GhRLCK24A*	*GhRLCK25D*
GhRLCK1A	*GhRLCK4D*	*GhRLCK41A*	*GhRLCK41D*	*GhRLCK56A*	*GhRLCK55D*
GhRLCK1A	*GhRLCK2D*	*GhRLCK26A*	*GhRLCK27A*	*GhRLCK56A*	*GhRLCK57D*
GhRLCK63A	*GhRLCK62A*	*GhRLCK26A*	*GhRLCK27D*	*GhRLCK70A*	*GhRLCK69D*
GhRLCK55A	*GhRLCK57A*	*GhRLCK23A*	*GhRLCK25A*	*GhRLCK52A*	*GhRLCK48D*
GhRLCK55A	*GhRLCK56A*	*GhRLCK23A*	*GhRLCK24A*	*GhRLCK52A*	*GhRLCK53D*
GhRLCK55A	*GhRLCK57D*	*GhRLCK23A*	*GhRLCK27D*	*GhRLCK52A*	*GhRLCK54D*
GhRLCK55A	*GhRLCK56D*	*GhRLCK23A*	*GhRLCK25D*	*GhRLCK52A*	*GhRLCK51D*
GhRLCK31A	*GhRLCK32A*	*GhRLCK23A*	*GhRLCK24D*	*GhRLCK15A*	*GhRLCK14D*
GhRLCK31A	*GhRLCK30A*	*GhRLCK36A*	*GhRLCK38D*	*GhRLCK1D*	*GhRLCK4D*
GhRLCK31A	*GhRLCK33D*	*GhRLCK36A*	*GhRLCK37A*	*GhRLCK1D*	*GhRLCK3D*
GhRLCK40A	*GhRLCK41A*	*GhRLCK6A*	*GhRLCK5A*	*GhRLCK1D*	*GhRLCK2D*
GhRLCK40A	*GhRLCK42D*	*GhRLCK6A*	*GhRLCK7A*	*GhRLCK11D*	*GhRLCK10D*
GhRLCK53A	*GhRLCK54A*	*GhRLCK6A*	*GhRLCK5D*	*GhRLCK11D*	*GhRLCK9D*
GhRLCK53A	*GhRLCK52A*	*GhRLCK6A*	*GhRLCK7D*	*GhRLCK32D*	*GhRLCK33D*
GhRLCK53A	*GhRLCK48D*	*GhRLCK67A*	*GhRLCK66D*	*GhRLCK41D*	*GhRLCK42D*
GhRLCK53A	*GhRLCK50D*	*GhRLCK32A*	*GhRLCK30A*	*GhRLCK53D*	*GhRLCK54D*
GhRLCK53A	*GhRLCK54D*	*GhRLCK32A*	*GhRLCK32D*	*GhRLCK53D*	*GhRLCK51D*
GhRLCK53A	*GhRLCK51D*	*GhRLCK62A*	*GhRLCK65A*	*GhRLCK53D*	*GhRLCK49D*
GhRLCK53A	*GhRLCK49D*	*GhRLCK62A*	*GhRLCK63D*	*GhRLCK66D*	*GhRLCK67D*
GhRLCK66A	*GhRLCK67A*	*GhRLCK62A*	*GhRLCK60D*	*GhRLCK28D*	*GhRLCK29D*
GhRLCK66A	*GhRLCK67D*	*GhRLCK5A*	*GhRLCK7A*	*GhRLCK60D*	*GhRLCK61D*
GhRLCK28A	*GhRLCK29D*	*GhRLCK5A*	*GhRLCK6D*	*GhRLCK48D*	*GhRLCK54D*
GhRLCK60A	*GhRLCK62A*	*GhRLCK5A*	*GhRLCK7D*	*GhRLCK48D*	*GhRLCK51D*
GhRLCK60A	*GhRLCK65A*	*GhRLCK7A*	*GhRLCK6D*	*GhRLCK48D*	*GhRLCK49D*
GhRLCK60A	*GhRLCK61D*	*GhRLCK7A*	*GhRLCK5D*	*GhRLCK30D*	*GhRLCK9D*
GhRLCK48A	*GhRLCK54A*	*GhRLCK7A*	*GhRLCK8D*	*GhRLCK54D*	*GhRLCK51D*
GhRLCK48A	*GhRLCK49A*	*GhRLCK30A*	*GhRLCK32D*	*GhRLCK54D*	*GhRLCK49D*
GhRLCK48A	*GhRLCK52A*	*GhRLCK30A*	*GhRLCK30D*	*GhRLCK50D*	*GhRLCK51D*
GhRLCK48A	*GhRLCK54D*	*GhRLCK9A*	*GhRLCK11D*	*GhRLCK4D*	*GhRLCK3D*
GhRLCK48A	*GhRLCK49D*	*GhRLCK9A*	*GhRLCK10D*	*GhRLCK14D*	*GhRLCK15D*
GhRLCK29A	*GhRLCK30A*	*GhRLCK65A*	*GhRLCK60D*	*GhRLCK37D*	*GhRLCK38D*
GhRLCK10A	*GhRLCK9A*	*GhRLCK25A*	*GhRLCK24A*	*GhRLCK6D*	*GhRLCK5D*
GhRLCK10A	*GhRLCK11D*	*GhRLCK25A*	*GhRLCK23D*	*GhRLCK6D*	*GhRLCK7D*
GhRLCK10A	*GhRLCK9D*	*GhRLCK25A*	*GhRLCK24D*	*GhRLCK5D*	*GhRLCK7D*
GhRLCK64A	*GhRLCK65A*	*GhRLCK27A*	*GhRLCK24A*	*GhRLCK7D*	*GhRLCK8D*
GhRLCK64A	*GhRLCK61D*	*GhRLCK27A*	*GhRLCK25D*	*GhRLCK25D*	*GhRLCK27D*
GhRLCK54A	*GhRLCK49A*	*GhRLCK27A*	*GhRLCK24D*	*GhRLCK25D*	*GhRLCK24D*

GhRLCK54A	*GhRLCK52A*	*GhRLCK57A*	*GhRLCK56A*	*GhRLCK27D*	*GhRLCK24D*
GhRLCK54A	*GhRLCK53D*	*GhRLCK57A*	*GhRLCK55D*	*GhRLCK57D*	*GhRLCK56D*
GhRLCK54A	*GhRLCK48D*	*GhRLCK57A*	*GhRLCK56D*	*GhRLCK69D*	*GhRLCK70D*

Table S4. Ka/KS calculation of the duplicated RLCK V-II genes in upland cotton

Paralogous pairs	Ka	Ks	Ka/Ks
GhRLCK8A/GhRLCK6A	0.079	0.54	0.147
GhRLCK8A/GhRLCK7A	0.078	0.475	0.163
GhRLCK8A/GhRLCK6D	0.082	0.524	0.157
GhRLCK8A/GhRLCK7D	0.078	0.481	0.161
GhRLCK1A/GhRLCK4A	0.286	2.015	0.142
GhRLCK1A/GhRLCK4D	0.264	2.126	0.124
GhRLCK1A/GhRLCK2D	0.223	0.681	0.328
GhRLCK63A/GhRLCK62A	0.534	4.99	0.107
GhRLCK55A/GhRLCK57A	0.086	0.5	0.172
GhRLCK55A/GhRLCK56A	0.068	0.479	0.142
GhRLCK55A/GhRLCK57D	0.085	0.492	0.173
GhRLCK55A/GhRLCK56D	0.067	0.507	0.133
GhRLCK31A/GhRLCK32A	0.108	0.406	0.267
GhRLCK31A/GhRLCK33D	0.099	0.407	0.244
GhRLCK40A/GhRLCK41A	0.058	0.469	0.124
GhRLCK40A/GhRLCK41A	0.053	0.469	0.114
GhRLCK53A/GhRLCK54A	0.081	0.61	0.132
GhRLCK53A/GhRLCK52A	0.117	1.128	0.103
GhRLCK53A/GhRLCK48D	0.297	1.303	0.228
GhRLCK53A/GhRLCK50D	0.234	1.729	0.135
GhRLCK53A/GhRLCK54D	0.083	0.582	0.143
GhRLCK53A/GhRLCK51D	0.242	2.206	0.11
GhRLCK53A/GhRLCK49D	0.284	1.745	0.163
GhRLCK66A/GhRLCK67A	0.093	0.505	0.184
GhRLCK66A/GhRLCK67D	0.09	0.508	0.176
GhRLCK28A/GhRLCK29D	0.077	0.676	0.115
GhRLCK60A/GhRLCK62A	0.173	1.67	0.103
GhRLCK60A/GhRLCK65A	0.177	3.067	0.058
GhRLCK60A/GhRLCK61D	0.069	0.698	0.099
GhRLCK48A/GhRLCK54A	0.282	1.865	0.151
GhRLCK48A/GhRLCK49A	0.113	0.641	0.177
GhRLCK48A/GhRLCK52A	0.343	1.561	0.219
GhRLCK48A/GhRLCK54D	0.281	1.854	0.151
GhRLCK48A/GhRLCK49D	0.112	0.659	0.17
GhRLCK29A/GhRLCK30A	0.098	0.77	0.127
GhRLCK10AGhRLCK9A	0.039	0.448	0.086
GhRLCK10A/GhRLCK11D	0.133	1.461	0.091
GhRLCK10A/GhRLCK9D	0.037	0.475	0.079
GhRLCK64A/GhRLCK65A	0.074	0.651	0.113
GhRLCK64A/GhRLCK61D	0.194	1.731	0.112
GhRLCK54A/GhRLCK49A	0.283	2.318	0.122
GhRLCK54A/GhRLCK52A	0.112	0.809	0.138
GhRLCK54A/GhRLCK53D	0.077	0.583	0.132
GhRLCK54A/GhRLCK48D	0.304	1.814	0.168
GhRLCK54A/GhRLCK51D	0.234	5.004	0.047
GhRLCK54A/GhRLCK49D	0.285	2.12	0.135
GhRLCK50A/GhRLCK53D	0.258	2.786	0.092
GhRLCK4A/GhRLCK1D	0.281	2.144	0.131
GhRLCK4A/GhRLCK3D	0.141	0.834	0.169

GhRLCK41A/GhRLCK42D	0.005	0.033	0.155
GhRLCK26A/GhRLCK27A	0.123	0.701	0.175
GhRLCK26A/GhRLCK27D	0.127	0.68	0.186
GhRLCK23A/GhRLCK25A	0.125	0.969	0.129
GhRLCK23A/GhRLCK24A	0.104	0.667	0.157
GhRLCK23A/GhRLCK27D	0.244	2.188	0.112
GhRLCK23A/GhRLCK25D	0.127	0.91	0.14
GhRLCK23A/GhRLCK24D	0.101	0.668	0.151
GhRLCK36A/GhRLCK38D	0.062	0.478	0.129
GhRLCK36A/GhRLCK37A	0.062	0.494	0.125
GhRLCK6A/GhRLCK5A	0.051	0.69	0.074
GhRLCK6A/GhRLCK7A	0.072	0.482	0.15
GhRLCK6A/GhRLCK5D	0.052	0.68	0.077
GhRLCK6A/GhRLCK7D	0.074	0.503	0.147
GhRLCK67A/GhRLCK66D	0.091	0.534	0.171
GhRLCK32A/GhRLCK32D	0.102	0.401	0.253
GhRLCK62A/GhRLCK65A	0.216	1.633	0.132
GhRLCK62A/GhRLCK63D	0.103	0.587	0.176
GhRLCK62A/GhRLCK60D	0.178	2.072	0.086
GhRLCK5A/GhRLCK7A	0.062	0.553	0.111
GhRLCK5A/GhRLCK6D	0.05	0.697	0.072
GhRLCK5A/GhRLCK7D	0.062	0.603	0.102
GhRLCK7A/GhRLCK6D	0.074	0.496	0.15
GhRLCK7A/GhRLCK5D	0.061	0.566	0.108
GhRLCK7A/GhRLCK8D	0.071	0.478	0.148
GhRLCK30A/GhRLCK30D	0.1	0.795	0.126
GhRLCK9A/GhRLCK11D	0.153	1.897	0.08
GhRLCK9A/GhRLCK10D	0.038	0.471	0.08
GhRLCK65A/GhRLCK60D	0.181	2.345	0.077
GhRLCK25A/GhRLCK24A	0.143	0.825	0.174
GhRLCK25A/GhRLCK23D	0.129	0.93	0.139
GhRLCK25A/GhRLCK24D	0.139	0.844	0.165
GhRLCK27A/GhRLCK24A	0.229	1.992	0.115
GhRLCK27A/GhRLCK24D	0.229	1.844	0.124
GhRLCK57A/GhRLCK56A	0.091	0.521	0.175
GhRLCK57A/GhRLCK55D	0.086	0.467	0.185
GhRLCK57A/GhRLCK56D	0.087	0.515	0.169
GhRLCK69A/GhRLCK70A	0.044	0.524	0.085
GhRLCK69A/GhRLCK70D	0.044	0.524	0.085
GhRLCK24A/GhRLCK23D	0.105	0.635	0.165
GhRLCK24A/GhRLCK27D	0.235	1.85	0.127
GhRLCK24A/GhRLCK25D	0.143	0.838	0.171
GhRLCK56A/GhRLCK55D	0.068	0.463	0.146
GhRLCK56A/GhRLCK57D	0.092	0.504	0.182
GhRLCK70A/GhRLCK69D	0.041	0.492	0.083
GhRLCK52A/GhRLCK48D	0.342	1.501	0.228
GhRLCK52A/GhRLCK53D	0.122	1.062	0.115
GhRLCK52A/GhRLCK54D	0.124	0.846	0.146
GhRLCK52A/GhRLCK51D	0.203	1.668	0.121
GhRLCK15A/GhRLCK14D	0.163	0.913	0.179
GhRLCK1D/GhRLCK4D	0.259	2.28	0.113

GhRLCK1D/GhRLCK3D	0.316	2.035	0.155
GhRLCK1D/GhRLCK2D	0.225	0.698	0.323
GhRLCK11D/GhRLCK10D	0.134	1.504	0.089
GhRLCK11D/GhRLCK9D	0.146	2.018	0.073
GhRLCK32D/GhRLCK33D	0.094	0.391	0.24
GhRLCK41D/GhRLCK42D	0.05	0.439	0.114
GhRLCK53D/GhRLCK54D	0.081	0.557	0.145
GhRLCK53D/GhRLCK51D	0.238	2.801	0.085
GhRLCK53D/GhRLCK49D	0.28	1.873	0.149
GhRLCK66D/GhRLCK67D	0.088	0.538	0.164
GhRLCK28D/GhRLCK29D	0.088	0.724	0.122
GhRLCK60D/GhRLCK61D	0.073	0.736	0.1
GhRLCK48D/GhRLCK54D	0.303	1.803	0.168
GhRLCK48D/GhRLCK51D	0.281	3.891	0.072
GhRLCK48D/GhRLCK49D	0.115	0.678	0.169
GhRLCK30D/GhRLCK9D	0.584	2.813	0.208
GhRLCK54D/GhRLCK51D	0.232	3.652	0.064
GhRLCK54D/GhRLCK49D	0.287	1.994	0.144
GhRLCK50D/GhRLCK51D	0.083	0.915	0.091
GhRLCK4D/GhRLCK3D	0.143	0.779	0.184
GhRLCK14D/GhRLCK15D	0.168	0.972	0.173
GhRLCK37D/GhRLCK38D	0.059	0.484	0.122
GhRLCK6D/GhRLCK5D	0.053	0.687	0.077
GhRLCK6D/GhRLCK7D	0.076	0.528	0.145
GhRLCK5D/GhRLCK7D	0.061	0.604	0.101
GhRLCK7D/GhRLCK8D	0.07	0.486	0.144
GhRLCK25D/GhRLCK27D	0.284	4.005	0.071
GhRLCK25D/GhRLCK24D	0.139	0.857	0.162
GhRLCK27D/GhRLCK24D	0.235	1.725	0.136
GhRLCK57D/GhRLCK56D	0.088	0.498	0.176
GhRLCK69D/GhRLCK70D	0.041	0.468	0.088

Table S5. The cis-elements in the promoter of *GhRLCKs*

Gene	MBS	LTR	TCA-element	CGTCA-motif	TGACG-motif	ABRE	P-box	GARE-motif	TATC-box	ERE	W-box	TC-rich repeats
GhRLCK8A	0	0	0	0	0	2	0	0	0	0	2	0
GhRLCK1A	0	0	0	0	0	0	0	0	0	1	0	1
GhRLCK22A	0	0	1	2	2	3	0	1	0	2	0	1
GhRLCK63A	0	0	1	0	0	3	0	0	0	6	0	1
GhRLCK11A	0	0	0	0	0	2	0	0	0	0	2	0
GhRLCK45A	0	0	2	2	2	3	0	0	0	1	1	1
GhRLCK59A	0	1	0	0	0	0	1	0	0	4	0	0
GhRLCK68A	0	0	0	0	0	0	0	0	0	1	0	0
GhRLCK58A	0	0	2	2	2	3	0	0	0	2	1	0
GhRLCK47A	1	0	0	1	1	0	0	0	1	2	0	1
GhRLCK46A	0	0	0	0	0	0	0	0	0	3	0	1
GhRLCK35A	0	1	2	0	0	2	0	0	0	0	0	0
GhRLCK55A	1	0	0	0	0	2	0	0	0	1	2	0
GhRLCK61A	0	0	1	0	0	2	0	0	0	1	0	0
GhRLCK31A	0	1	0	1	1	0	0	0	0	1	0	1
GhRLCK33A	1	0	0	0	0	0	1	0	0	5	0	0
GhRLCK40A	2	0	1	0	0	1	0	0	0	4	0	0
GhRLCK53A	1	0	0	1	1	4	0	0	0	5	1	1
GhRLCK66A	0	0	1	0	0	0	0	0	0	2	1	0
GhRLCK28A	1	0	0	0	0	1	0	0	0	7	0	0
GhRLCK60A	0	0	1	1	1	0	0	0	0	3	0	0
GhRLCK48A	1	2	2	1	1	1	0	0	0	0	0	1
GhRLCK29A	0	0	1	0	0	3	0	0	0	3	0	0
GhRLCK10A	1	0	0	0	0	0	0	0	0	6	0	2
GhRLCK64A	0	0	0	0	0	5	0	0	0	1	0	1
GhRLCK19A	0	0	1	5	5	2	0	0	0	1	0	0
GhRLCK42A	1	0	0	2	2	0	2	0	0	0	0	0
GhRLCK54A	0	0	0	1	1	3	0	0	0	0	0	0
GhRLCK50A	0	0	0	1	1	0	0	0	0	4	2	0
GhRLCK4A	0	0	0	0	0	0	0	0	0	4	0	0
GhRLCK41A	0	0	0	0	0	1	0	0	0	3	2	1
GhRLCK51A	0	0	0	2	2	3	0	0	0	2	1	1
GhRLCK44A	0	1	1	1	1	1	0	0	0	2	0	0
GhRLCK26A	0	0	0	2	2	0	0	0	0	6	2	0
GhRLCK23A	1	0	1	3	3	5	0	0	0	1	0	0
GhRLCK14A	2	0	1	2	2	2	1	0	0	1	2	0
GhRLCK36A	0	1	0	0	0	1	1	0	0	5	1	0
GhRLCK21A	0	0	0	0	0	0	0	0	0	0	0	0
GhRLCK6A	0	0	1	0	0	0	0	0	0	1	0	0
GhRLCK67A	2	1	0	1	1	1	2	0	0	0	3	0
GhRLCK32A	0	0	0	0	0	0	0	0	0	1	0	0

GhRLCK62A	0	0	0	0	0	0	0	0	0	0	0	0
GhRLCK5A	0	0	0	0	0	0	0	0	0	0	0	0
GhRLCK49A	0	0	1	0	0	0	1	0	0	3	0	0
GhRLCK7A	0	0	1	0	0	0	0	0	0	0	0	0
GhRLCK30A	0	0	1	1	1	0	1	0	0	1	0	0
GhRLCK9A	0	0	2	0	0	0	0	0	0	7	0	0
GhRLCK65A	0	0	0	0	0	0	2	0	0	2	1	0
GhRLCK25A	2	0	1	0	0	1	2	0	0	2	0	0
GhRLCK20A	0	0	0	0	0	0	0	1	0	3	0	1
GhRLCK27A	1	0	0	3	3	1	1	0	1	0	3	0
GhRLCK16A	0	0	0	1	1	1	0	0	0	2	1	0
GhRLCK17A	0	1	1	1	1	1	0	0	0	0	2	0
GhRLCK37A	0	0	1	1	1	2	1	0	0	1	0	0
GhRLCK57A	1	0	0	2	2	0	0	0	0	3	2	0
GhRLCK69A	0	1	0	1	1	2	0	0	0	5	1	0
GhRLCK24A	0	1	2	1	1	1	0	0	0	0	2	1
GhRLCK38A	0	1	0	0	0	1	0	0	0	3	1	0
GhRLCK56A	3	1	0	0	0	3	1	0	0	1	3	0
GhRLCK70A	0	0	2	0	0	2	0	0	0	4	1	0
GhRLCK18A	0	0	1	0	0	0	0	0	0	0	1	2
GhRLCK52A	0	0	1	3	3	1	0	1	1	5	1	2
GhRLCK43A	0	0	1	0	0	2	0	0	0	3	1	0
GhRLCK15A	0	1	0	2	2	2	1	1	0	1	3	0
GhRLCK13D	0	0	2	0	0	0	1	0	0	1	1	1
GhRLCK12D	1	2	2	2	2	1	0	0	0	3	0	1
GhRLCK1D	0	0	0	0	0	0	0	0	0	1	1	0
GhRLCK22D	0	0	1	2	2	3	1	1	0	0	0	1
GhRLCK35D	0	0	3	3	3	3	1	0	0	2	0	1
GhRLCK11D	0	1	3	0	0	1	0	0	0	2	1	0
GhRLCK63D	1	0	1	0	0	2	0	0	0	5	0	0
GhRLCK8D	1	0	0	0	0	1	0	0	0	0	2	0
GhRLCK45D	0	1	1	1	1	0	0	0	0	1	0	0
GhRLCK36D	0	0	1	1	1	4	1	0	0	1	0	0
GhRLCK55D	1	1	0	1	1	2	0	0	0	1	2	0
GhRLCK58D	0	0	2	2	2	4	0	0	0	1	1	0
GhRLCK68D	0	1	0	0	0	0	0	0	0	2	0	0
GhRLCK46D	0	1	0	0	0	0	0	0	0	0	0	2
GhRLCK47D	1	0	0	1	1	0	0	0	0	8	0	0
GhRLCK43D	1	1	1	2	3	0	2	0	0	1	1	0
GhRLCK19D	1	1	1	1	1	0	1	0	0	3	0	0
GhRLCK40D	0	0	2	0	0	1	2	0	0	0	0	2
GhRLCK32D	0	3	1	1	1	0	0	0	0	1	1	0
GhRLCK34D	1	0	0	0	0	1	1	0	0	3	0	0
GhRLCK41D	0	0	1	1	1	1	0	0	0	3	1	0

GhRLCK53D	2	1	0	1	1	4	1	0	0	2	0	1
GhRLCK66D	0	1	2	0	0	1	0	0	0	2	1	0
GhRLCK28D	0	0	0	1	1	0	0	0	0	8	0	0
GhRLCK60D	1	0	1	1	1	0	0	0	0	2	0	0
GhRLCK48D	2	1	2	1	1	1	0	0	0	0	0	1
GhRLCK30D	0	0	1	0	0	4	0	0	0	5	0	0
GhRLCK10D	1	0	0	0	0	0	1	0	0	5	0	1
GhRLCK54D	0	0	1	2	2	6	0	0	0	0	1	0
GhRLCK50D	1	0	0	0	0	0	0	0	0	1	0	0
GhRLCK4D	0	2	0	1	1	0	0	1	0	3	0	1
GhRLCK51D	0	0	1	1	1	5	0	0	0	1	1	0
GhRLCK42D	0	0	1	1	1	0	0	0	0	3	1	1
GhRLCK14D	2	1	0	0	0	2	1	0	0	1	2	0
GhRLCK23D	1	0	2	5	5	4	0	0	0	1	0	0
GhRLCK37D	0	0	1	0	0	0	0	0	0	2	0	0
GhRLCK21D	1	1	0	0	0	2	1	0	0	0	1	0
GhRLCK6D	1	0	1	0	0	0	0	0	0	4	1	0
GhRLCK67D	1	0	0	1	1	1	2	0	0	0	2	0
GhRLCK33D	0	1	1	2	2	3	0	0	0	1	0	1
GhRLCK5D	0	0	0	0	0	0	0	0	0	0	0	0
GhRLCK3D	0	0	0	0	0	0	0	0	0	0	0	0
GhRLCK49D	0	0	0	0	0	0	0	0	0	0	0	0
GhRLCK7D	0	1	0	0	0	0	2	0	0	5	0	0
GhRLCK9D	0	0	3	0	0	0	0	0	0	6	0	0
GhRLCK31D	0	0	0	3	3	1	1	0	0	0	0	0
GhRLCK25D	1	0	0	0	0	1	2	0	0	3	0	0
GhRLCK20D	0	0	0	1	1	0	0	1	0	4	0	1
GhRLCK38D	0	0	1	0	0	2	1	1	0	0	0	0
GhRLCK27D	0	0	0	3	3	2	1	0	1	0	3	1
GhRLCK16D	0	1	1	0	0	1	0	0	0	2	2	0
GhRLCK2D	1	0	1	0	0	2	1	0	1	0	0	0
GhRLCK17D	0	0	0	0	0	1	0	0	0	3	2	0
GhRLCK57D	1	0	0	0	0	1	0	0	0	2	2	0
GhRLCK69D	0	1	0	3	3	4	1	0	0	2	0	0
GhRLCK24D	2	1	2	0	0	0	0	0	0	6	1	0
GhRLCK56D	3	0	0	1	1	2	1	0	0	1	2	0
GhRLCK39D	0	1	0	0	0	1	0	0	0	3	1	0
GhRLCK70D	0	1	0	1	1	0	0	0	1	0	0	1
GhRLCK18D	0	1	2	0	0	1	0	0	0	0	1	1
GhRLCK44D	0	0	1	1	1	0	0	0	0	2	0	1
GhRLCK29D	2	0	2	0	0	0	0	0	0	4	1	0
GhRLCK61D	0	0	1	1	1	2	1	0	0	1	0	1
GhRLCK15D	0	1	0	2	2	2	1	1	0	0	3	0
GhRLCK52D	0	0	1	3	3	1	0	1	1	4	1	0

Table S6. Primers used in this study

Gene	Forward Primer	Reverse Prime
**RT-PCR primers**		
GhRLCK18A	CGTCAAGAATGCTTGCTG	ATGCTGTAAGAGGCTCAG
GhRLCK7	GGTCGTTATCCCATACGA	GTCTGCCATACTCCTTCG
GhRLCK1A	CGGCATCATTGGAGAAGAGC	GCTGATTCTCCGGGTTGAAG
GhRLCK20A	GAAACCCAAGCCTCCTGAATC	ATCCACCTTGCCCGATCATG
GhRLCK24A	CTGCTCCTGAACTGAGAAAC	TGTGTGGCATCCCTCAACTC
GhRLCK54D	TGGAGATCCATCATCCCTAG	AGGTAGACAGATCCTCCGAC
GhRLCK53D	AACAAGACGAGGACACCGTC	CTATGATTTCGCTTCAGGGC
GhRLCK4D	GTCTTGTTGTAGGTCGGATG	ATCTTCTGCTTGCCTCCATC
GhRLCK49	ACCCAAGGATTCATCTTCCG	ATCACTCCTAGTTCCTTGAG
GhRLCK64A	GCTAGAATCAAAGCTGAGAGC	GTGGCTGTTCTAAGTTCACTG
GhRLCK9A	ACACCACCACCAACAACAAC	GTTGAAGCTGCCAATTCACG
GhRLCK43A	ATTGAAGAAGAGCCGGAC	GGATTCTGCCGTAGATCTTTG
GhRLCK22D	TCGCCATTCTGCTGATTCG	CAAATCCTCCTTCACCAAGC
GhRLCK42D	TGTTGGGCTGTCTTGAAACG	TGGATCAGGGTTGTCAGATG
GhUBQ7	AGGCATTCCACCTGACCAAC	CAGCGAGCTTGACCTTCTTC
**VIGS primer**		
GhRLCK7	CGGAATTCGGCCAGAATGTCTATTAGG	GGGGTACCTTTGTCACCAACAGGACC
